# Supplementary figures and images for: Fusing an agent-based model of mosquito population dynamics with a statistical reconstruction of spatio-temporal abundance patterns
Source: PLoS Comput Biol. 2023 Apr 27;19(4):e1010424. doi: 10.1371/journal.pcbi.1010424 (PMC10168549; doi:10.1371/journal.pcbi.1010424)

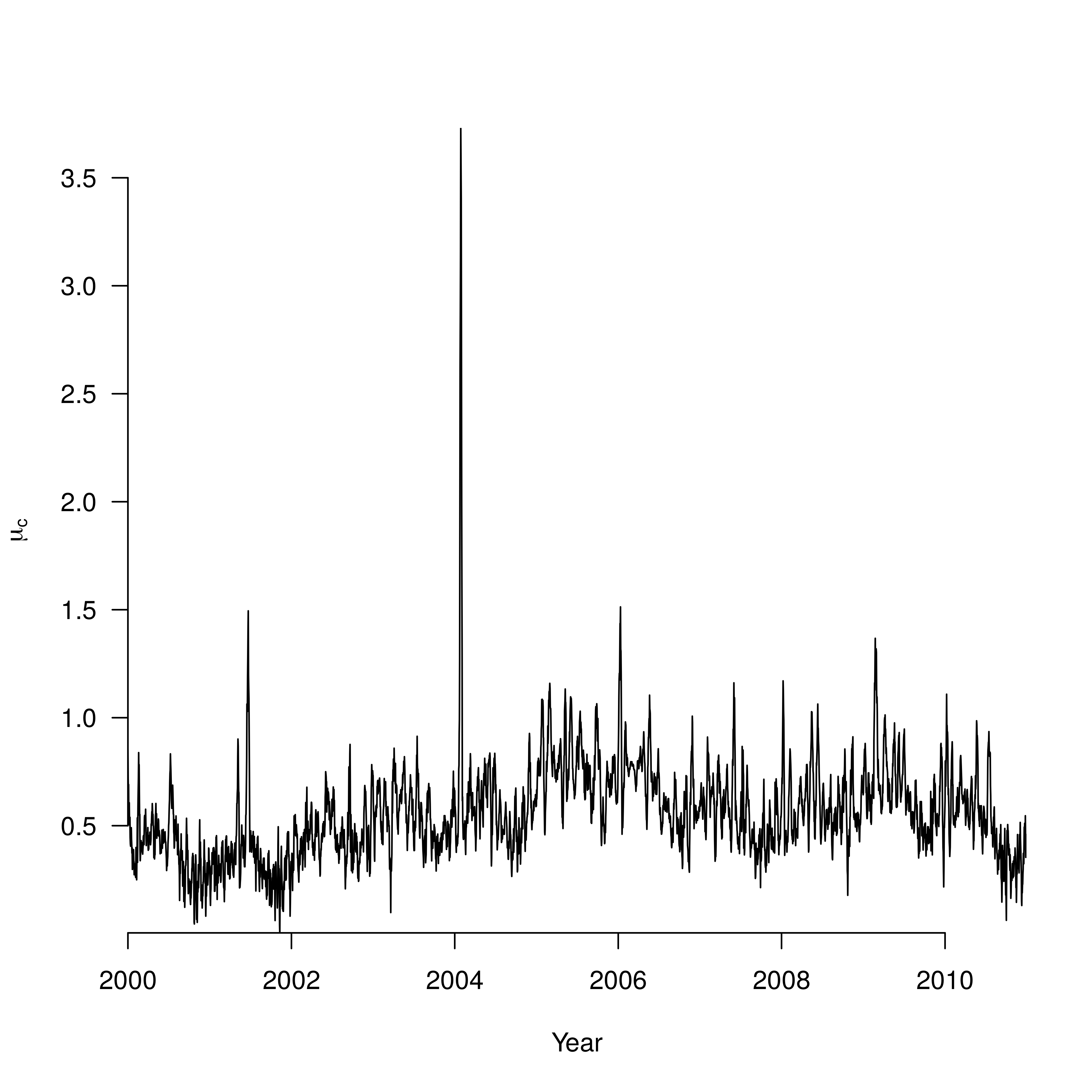

Supplement: S1 Fig — This parameter forms a key step in our approach, linking the mechanistic and statistical models, and can be thought of as accoutning for other sources of mortality that are not captured by the temperature-mortality relationships. The parameter is always positive (i.e. it is always a mortality rate). (TIF) [file pcbi.1010424.s001.tif]

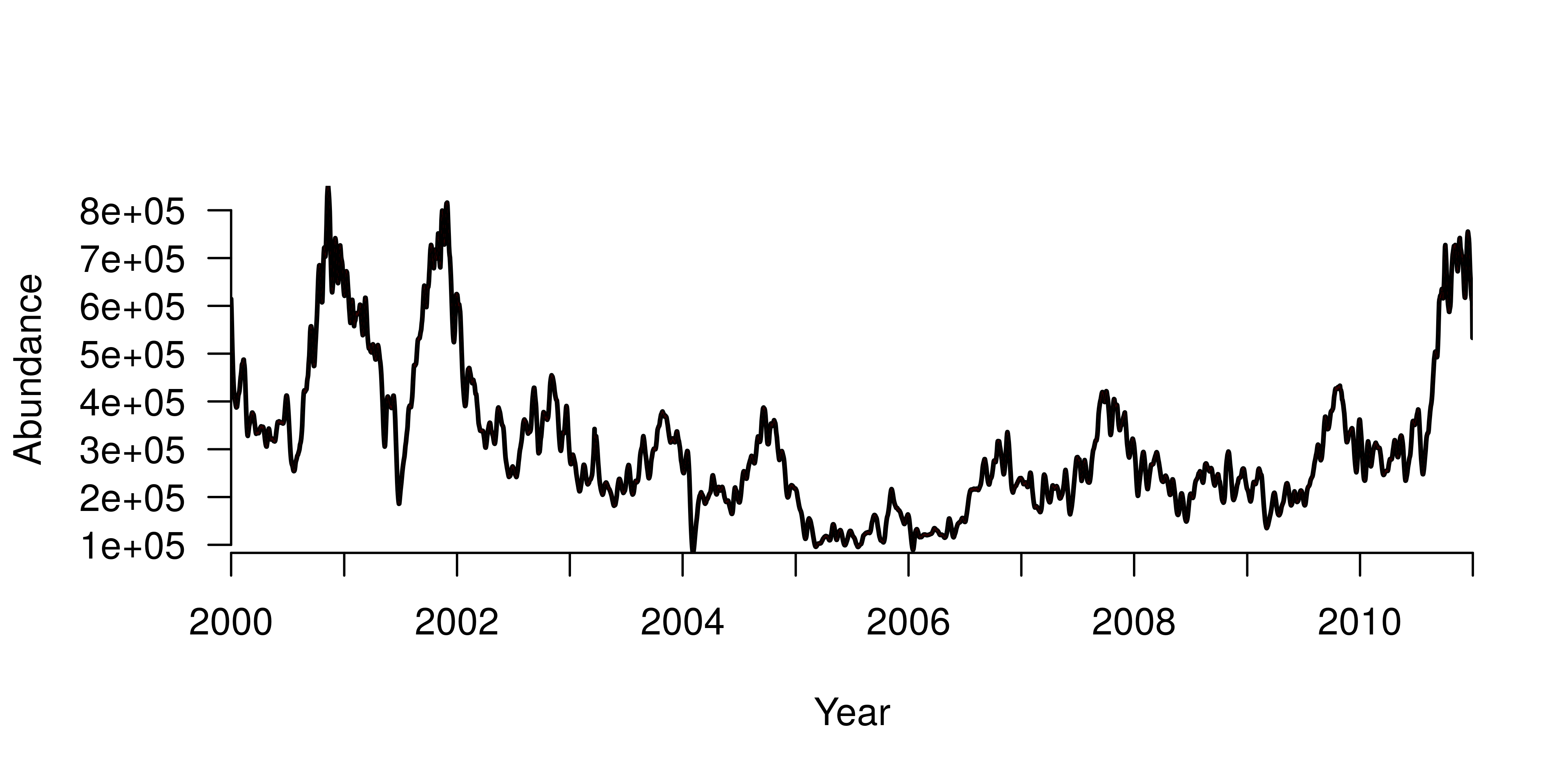

Supplement: S2 Fig — Due to the lack of stochasticity in the mosquito component of the model, there is little variability between runs, and so the 95% CI is indistinguishable from the mean. (TIF) [file pcbi.1010424.s002.tif]

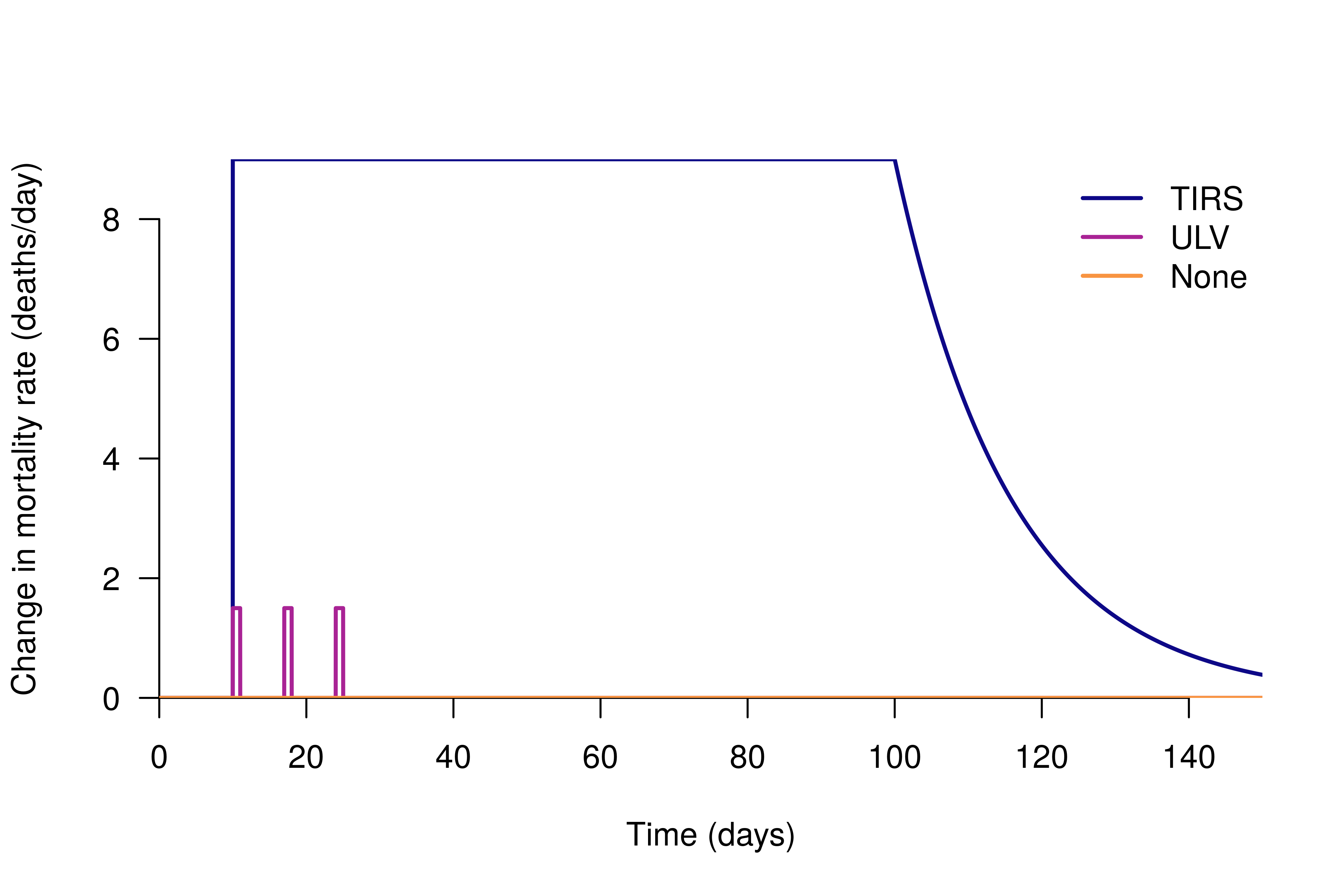

Supplement: S3 Fig — In this example, there was one targeted insecticide residual spraying (TIRS) campaign, which began on day 10, three ultra-low volume (ULV) campaigns, or neither, which began on days 10, 17, and 24. The mortality rate increases by the shown amount. (TIF) [file pcbi.1010424.s003.tif]

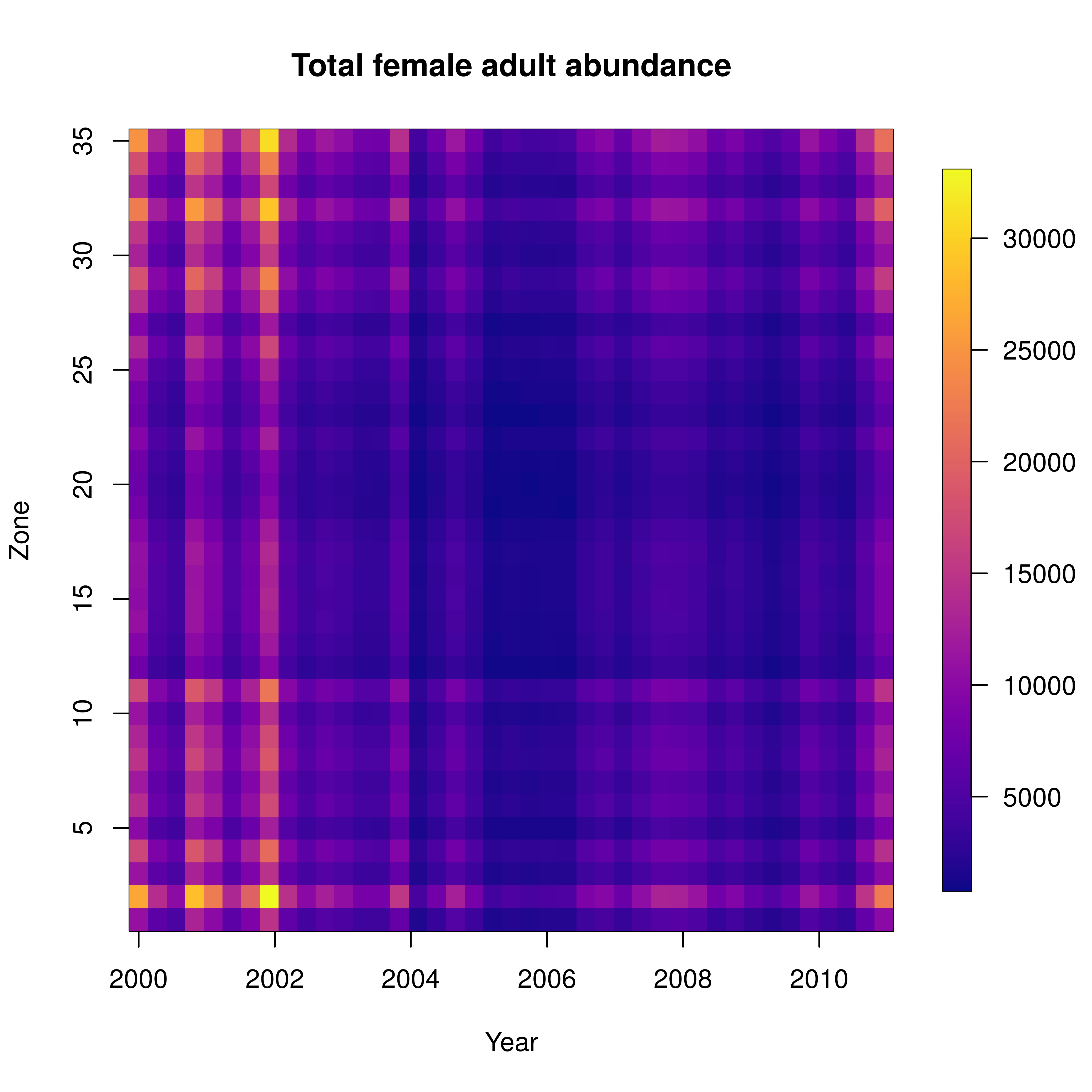

Supplement: S4 Fig — As in Fig 4, but not normalized by the total population (i.e., the total zonal abundance). Each column represents the daily abundance every 100 days from 2000–2010. Each row is a Ministry of Health zone in Iquitos. Columns are normalized by the total abundance that day. (TIF) [file pcbi.1010424.s004.tif]

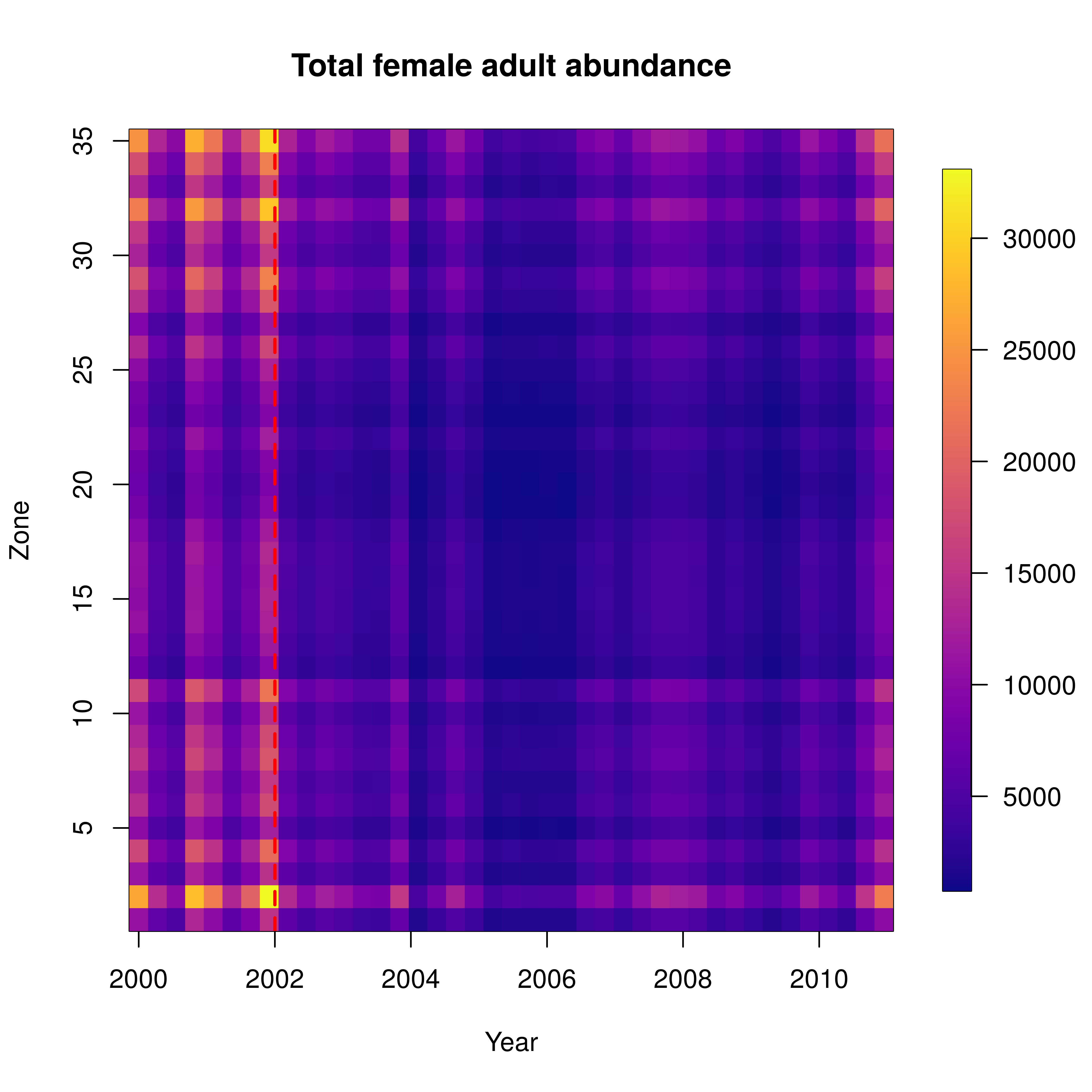

Supplement: S5 Fig — As in Fig 6, but not normalized by the total population (i.e., the total zonal abundance). Each column represents the daily abundance every 100 days from 2000–2010. Each row is a Ministry of Health zone in Iquitos. Columns are normalized by the total abundance that day. (TIF) [file pcbi.1010424.s005.tif]

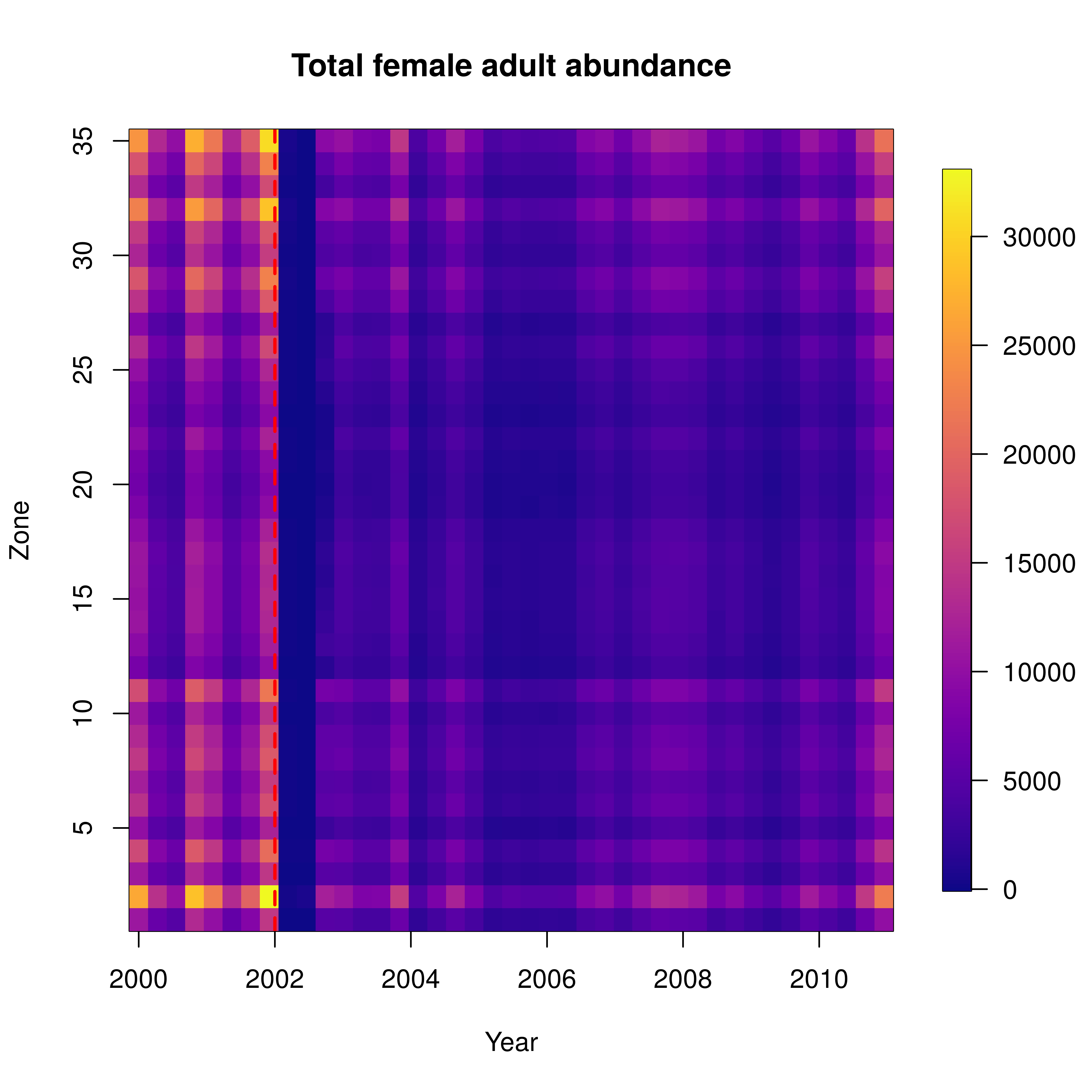

Supplement: S6 Fig — As in Fig 7, but not normalized by the total population (i.e., the total zonal abundance). Each column represents the daily abundance every 100 days from 2000–2010. Each row is a Ministry of Health zone in Iquitos. Columns are normalized by the total abundance that day. (TIF) [file pcbi.1010424.s006.tif]

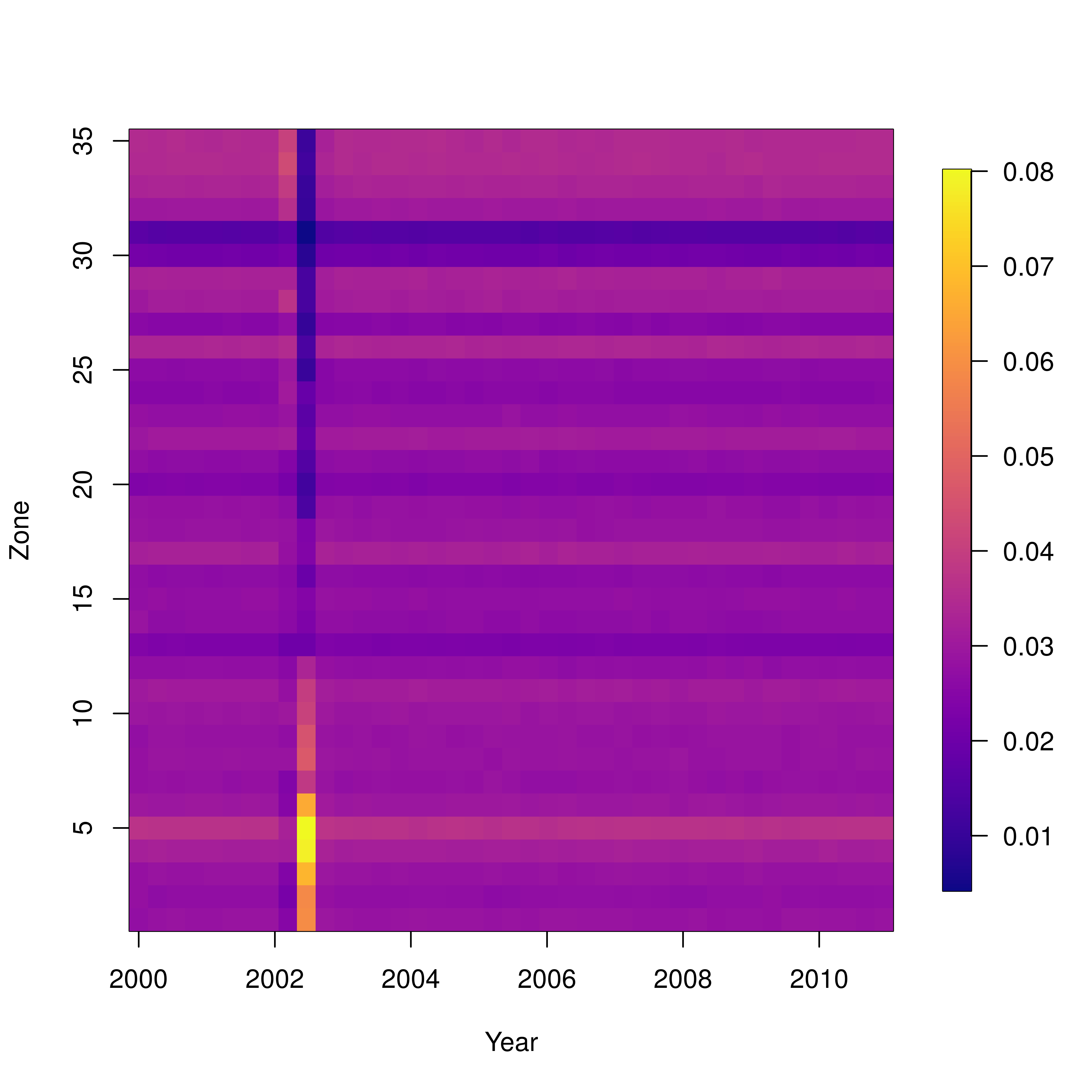

Supplement: S7 Fig — Normalized mosquito abundance over time, following a city-wide TIRS campaign initiated on the 1st January 2002, indicated by the red dashed line. Each column represents the daily abundance every 100 days from 2000–2010. Each row is a Ministry of Health zone in Iquitos. Columns are normalized by the total abundance that day. (TIF) [file pcbi.1010424.s007.tif]

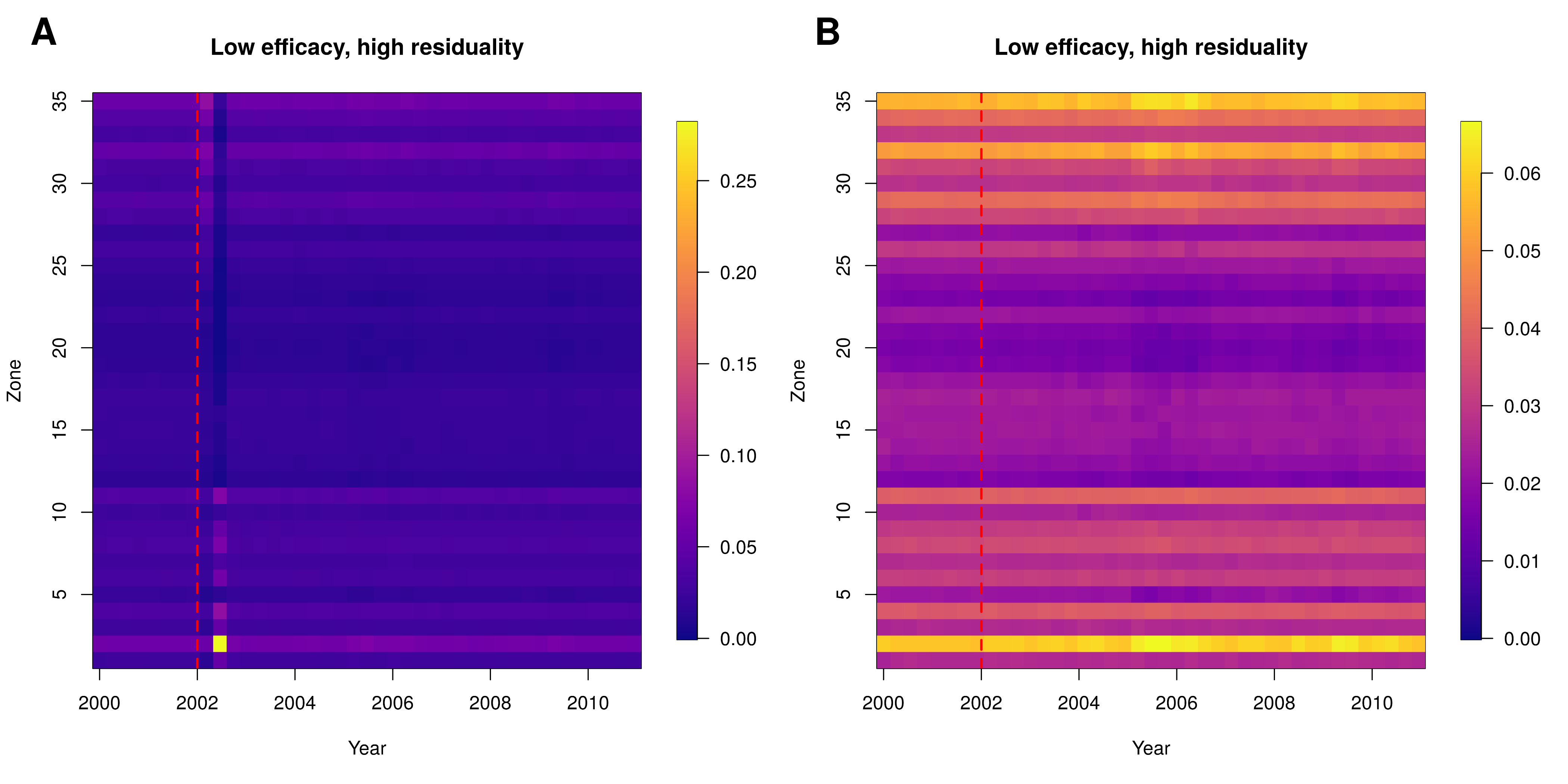

Supplement: S8 Fig — a city-wide campaign with a hypothetical insecticide that causes a large increase in mortality (equivalent to TIRS) with low residuality (equivalent to ULV) and B. city-wide campaign with a hypothetical insecticide that causes a small increase in mortality (equivalent to ULV) with high residuality (equivalent to TIRS). (TIF) [file pcbi.1010424.s008.tif]

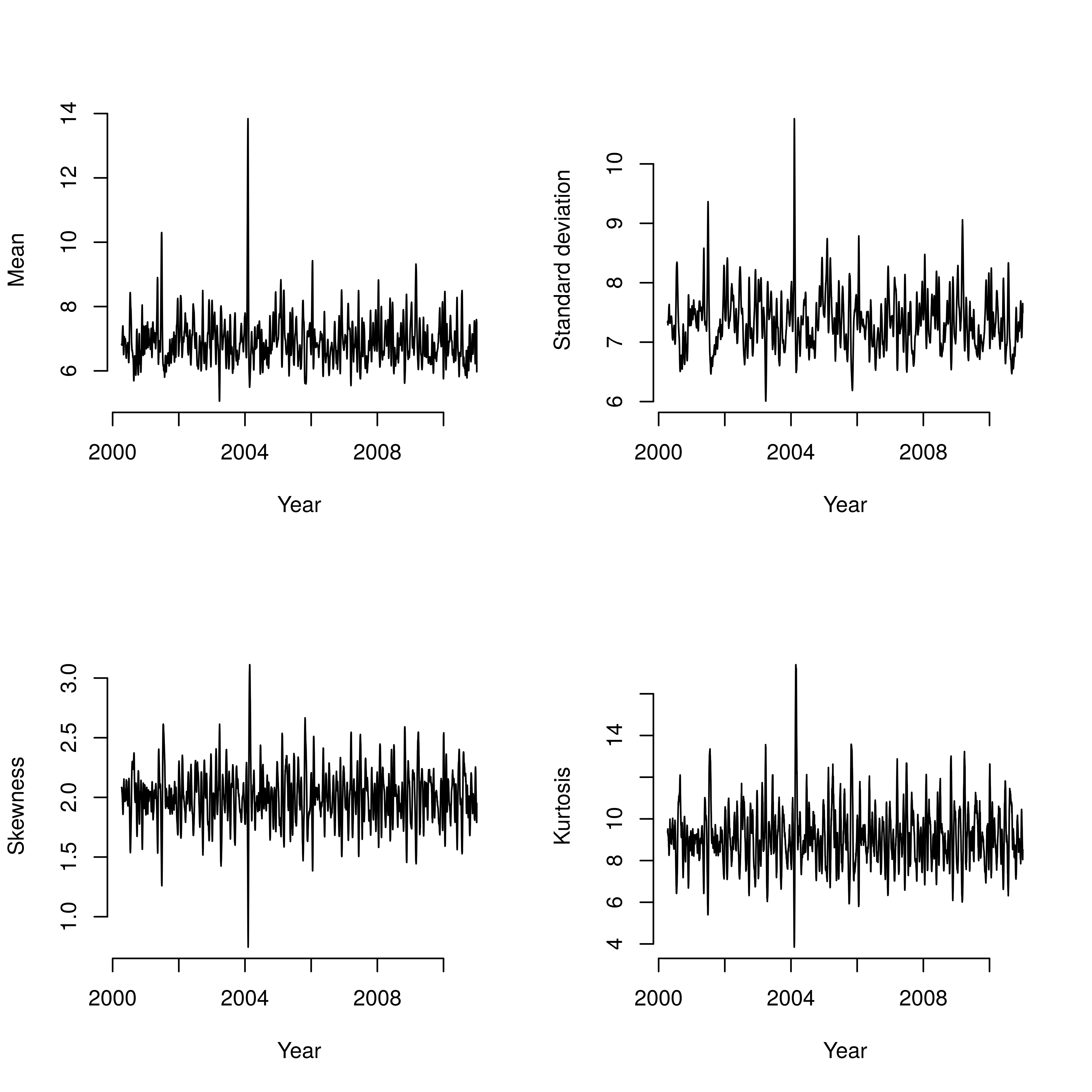

Supplement: S9 Fig — In early 2004, a cohort of mosquitoes reaches an older age, increasing all of the moments as the average age increased and the distribution becomes more skewed and bimodal. (TIF) [file pcbi.1010424.s009.tif]

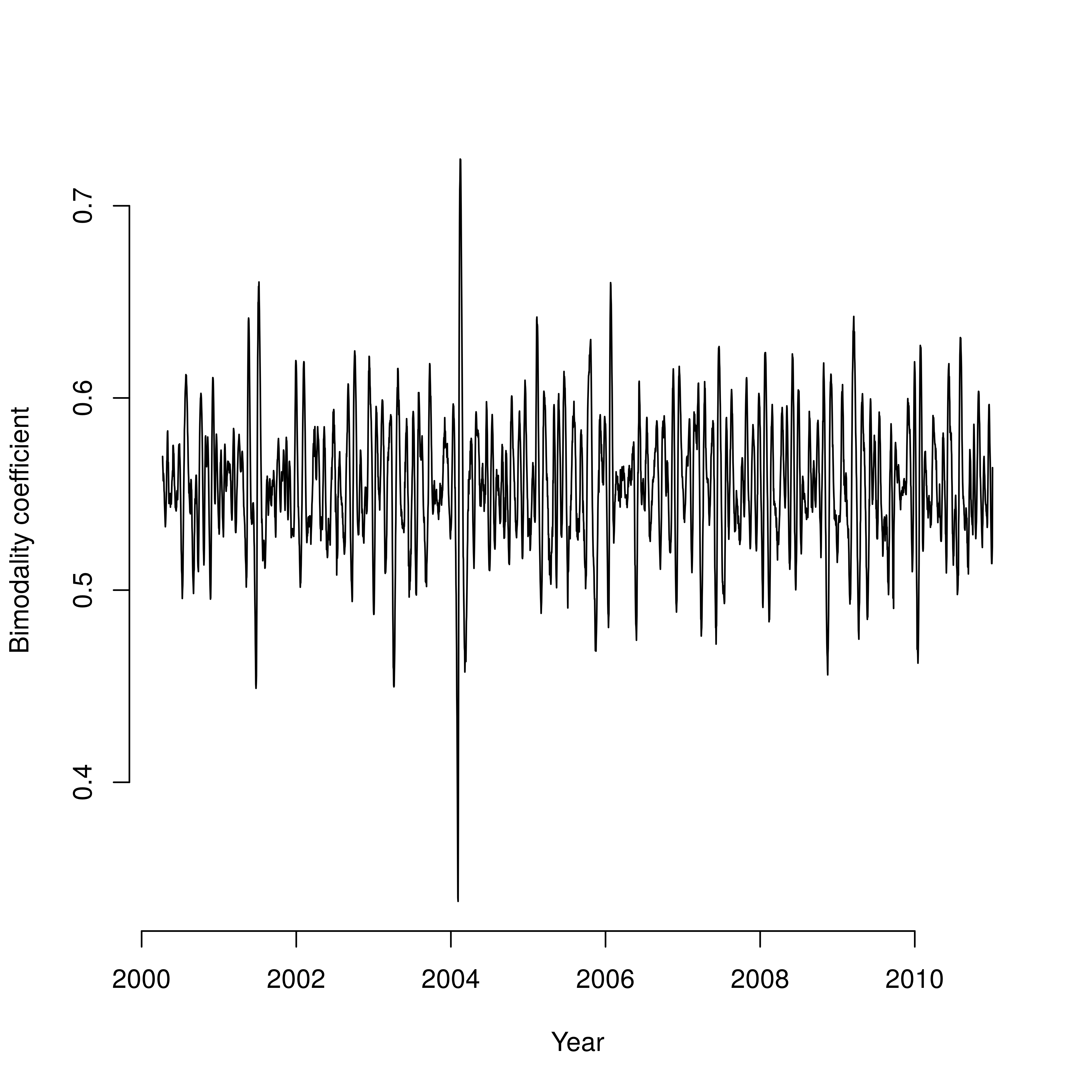

Supplement: S10 Fig — The bimodality coefficient takes a value between 0 and 1, and higher values mean the distribution is ‘more’ bimodal. It is defined as γ2+1κ, where γ is the skewness and κ is the kurtosis. In early 2004, a cohort of mosquitoes reached an older age, resulting in a bimodal age distribution. (TIF) [file pcbi.1010424.s010.tif]

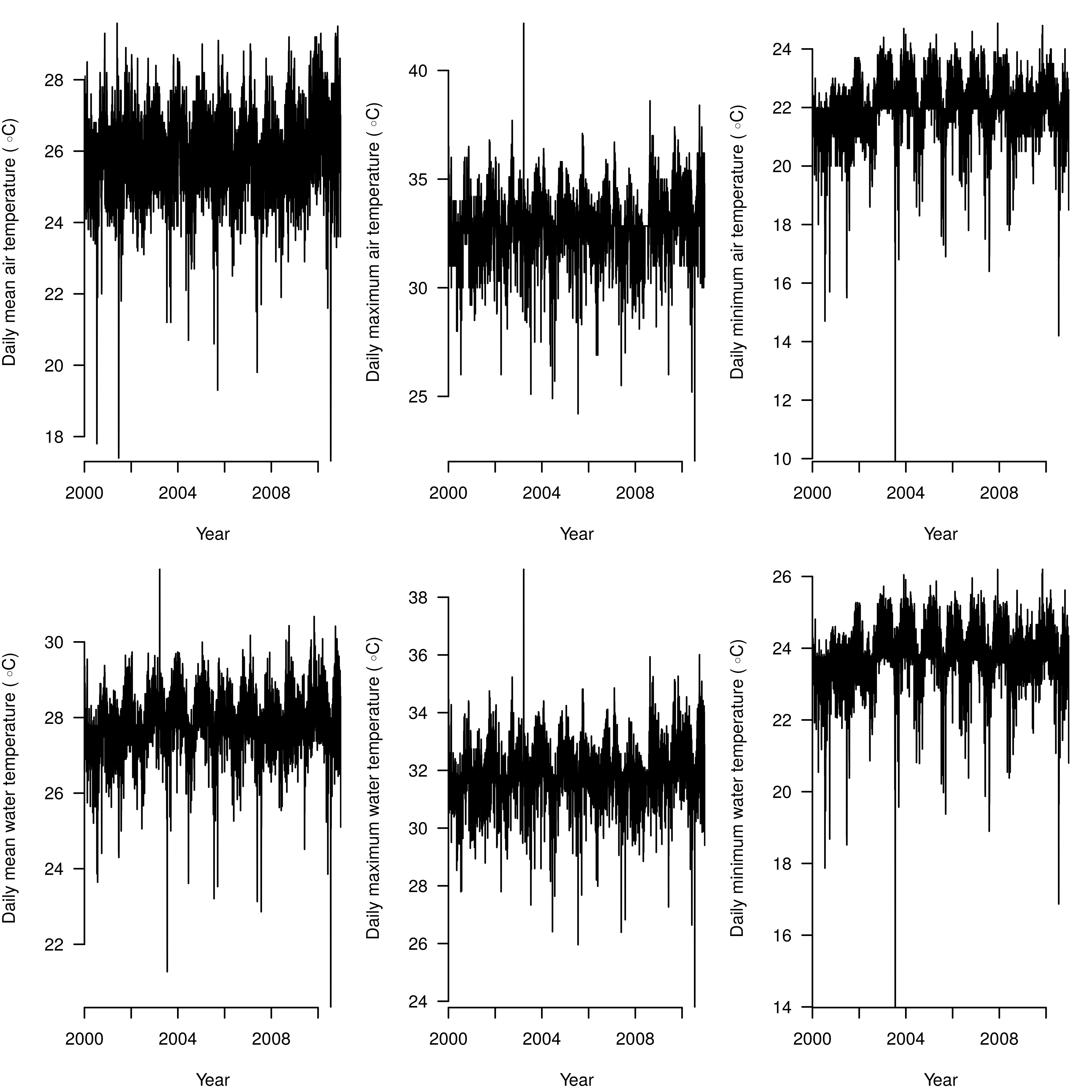

Supplement: S11 Fig — (TIF) [file pcbi.1010424.s011.tif]
