# Supplementary material for: Fusing an agent-based model of mosquito population dynamics with a statistical reconstruction of spatio-temporal abundance patterns
Source: PLoS Comput Biol. 2023 Apr 27;19(4):e1010424. doi: 10.1371/journal.pcbi.1010424 (PMC10168549; doi:10.1371/journal.pcbi.1010424)
Supplement: S1 Text — (DOCX) [file pcbi.1010424.s012.docx]

### S1 Text

- 1. We parameterize the transitions in the early life-stages in a way analagous to that of Magori et al. [1] Although there are more recent estimates of the thermal responses of *Aedes aegypti* life traits, such as Mordecai et al. [2], we wished to retain comparability with prior agent-based models of *Ae. aegypti* population dynamics and so used the same parameterization as Magori et al. This parameterization is as follows:

Water temperature is a function of the daily minimum air temperature (*T_min_*), daily maximum air temperature (*T_max_*), and average sun exposure (*S*), expressed as a proportion [1]. In the absence of data on sun exposure, we set *S=*0.1 in all simulations. Water temperature is then given by:

- 1. The development rate of mosquito life stage *i* as a function of temperature (water temperature for immature stages, and air temperature for mature stages) is
  $d_{i}\left( T \right)=\frac{\rho_{i}\frac{T}{298}exp\left( \frac{\Delta_{A,i}}{R}\left( \frac{1}{298}-\frac{1}{T_{W,mean}} \right) \right)}{1+exp\left( \frac{\Delta_{H,i}}{R}\left( \frac{1}{T_{1/2}}-\frac{1}{T_{W,mean}} \right) \right)}$
  2. where the stage-specific parameters are given in S1 Table [1].
  3. S1 Table. Parameters of the temperature-dependent enzyme-kinetics developmental rate model, from Magori et al. [1]

| ***Parameter*** | ***Definition*** | ***Eggs*** | ***Larvae*** | ***Pupae*** | ***Gonotrophic cycle*** |
| --- | --- | --- | --- | --- | --- |
| $\rho_{i}$ | Development rate per hour at 25ºC assuming no temperature inactivation of the critical enzyme (hr^-1^) | 0.01066 | 0.00873 | 0.01610 | 0.00898 |
| $\Delta_{A,i}$ | Enthalpy of activation of the reaction catalyzed by the enzyme (cal/mol) | 10,798.18 | 26,018.51 | 14,931.94 | 15,725.23 |
| $\Delta_{H,i}$ | Enthalpy change associated with high temperature inactivation of the enzyme (cal/mol) | 100,000 | 55,990.75 | -472,379 | 1,756,481.07 |
| $T_{1/2}$ | Temperature at which 50% of the enzyme is inactivated from high temperature | 14184.5 | 304.58 | 148.45 | 447.17 |

- 1. The mortality rate as a function of temperature is $\mu\left( T_{max} \right)=-log\left( s_{0}s_{T}\left( T_{max} \right) \right)$. Here, *s_0_* is the nominal daily survival probabilty and $s_{T}\left( T_{max} \right)$ is the reduction in survival probability caused by excessively warm temperatures. Values for these are given in S2 Table. Mortality effects from cold temperatures begin to occur at water temperatures of 10°C and air temperatures of 4°C [1]. In Iquitos the temperature never gets below these levels, so only additional mortality from excessively warm temperatures is considered. See S11 Fig for the temperature time series for air and water in Iquitos in the period 2000-2010. The temperature dependent survival is
  2. $s_{T}\left( T_{max} \right)=1$ if $T_{max}<T_{0}$,
  3. $s_{T}\left( T_{max} \right)=1-0.95\frac{T_{max}-T_{0}}{T_{\infty}-T_{0}}$ if $T_{0}<T_{max}<T_{\infty}$, and
  4. $s_{T}\left( T_{max} \right)=0.05$, if $T_{max}>T_{\infty}$.

1. S2 Table. Parameters for daily mortality functions

| ***Parameter*** | ***Eggs*** | ***Larvae*** | ***Pupae*** | **Adults** |
| --- | --- | --- | --- | --- |
| Nominal daily survival, *s_0_* | 0.99 | 0.99 | 0.77 | 0.89 |
| *T_0_* | 30 | 39 | 39 | 40 |
| *T_∞_* | 47 | 44 | 44 | 50 |

**References**

1. Magori K, Legros M, Puente ME, Focks DA, Scott TW, Lloyd AL, et al. Skeeter Buster: a stochastic, spatially explicit modeling tool for studying Aedes aegypti population replacement and population suppression strategies. PLoS Negl Trop Dis. 2009;3: e508. doi:10.1371/journal.pntd.0000508

2. Mordecai EA, Cohen JM, Evans MV, Gudapati P, Johnson LR, Lippi CA, et al. Detecting the impact of temperature on transmission of Zika, dengue, and chikungunya using mechanistic models. PLOS Neglected Tropical Diseases. 2017;11: e0005568. doi:10.1371/journal.pntd.0005568
