# Supplementary material for: Fusing an agent-based model of mosquito population dynamics with a statistical reconstruction of spatio-temporal abundance patterns
Source: PLoS Comput Biol. 2023 Apr 27;19(4):e1010424. doi: 10.1371/journal.pcbi.1010424 (PMC10168549; doi:10.1371/journal.pcbi.1010424)
Supplement: S2 Text — (DOCX) [file pcbi.1010424.s013.docx]

**S2 Text**

Our agent-based model (ABM) includes both people and mosquitoes as agents. These agents interact with each other in an environment represented by a set of locations, including houses, schools, parks, cemeteries, and churches. This environment also incorporates temporally varying climatic conditions, which affect mosquito biting frequency, survival, and extrinsic incubation period for dengue virus (DENV). The environment is based on the city of Iquitos in Peru, and we represent all 92,891 buildings in the city. We use exact spatial coordinates and location type for the 38,835 locations for which these data were available. For locations without these data, we randomly distributed the locations and assigned a location type so that they were evenly spaced and representative of the location types we had data on. In this study, we only used the mosquito population dynamics and mosquito movement aspects of the model.

Immature mosquitoes were modeled deterministically and independently at each location. They transitioned through three immature stages: eggs, larvae, and pupae, with the number of pupae in a house determining the rate of emergence of adult mosquitoes in that house. The rate of transition between each of these stages was temperature dependent. All stages also underwent temperature-dependent mortality. Larval stages underwent an additional density-dependent mortality [1,2]. Both the larval and pupal stages also underwent an additional rate of mortality that was calibrated so that adult abundance matched a statistical estimate of the spatio-temporal adult abundance in Iquitos [3]. Adult mosquitoes are modeled as agents, and take blood-meals upon co-located human agents. When a mosquito takes a blood-meal, the time of its next blood-meal is determined according to an exponential distribution with a temperature-dependent gonotrophic rate parameter, based on temporal trends in temperature and the empirical relationships between these rates and temperature described in S1 Text [2,4]. When the mosquito’s next blood-meal is due, it will take it unless there is no human present. This means that the number of blood-meals taken by a mosquito is not determined by the local density of humans, except in the unusual instance in which no human is present at a location. The mosquito determines which human it will bite as a function of the body sizes of humans present at that time – the mosquito chooses who to bite with probability proportional to the surface-area of individuals in the building at that time [5]. Each day, each mosquito moves to another location with probability 0.3, and will only move to a location within 100 m of its starting location, consistent with another agent-based model of *Ae. aegypti* population dynamics [1].

*References*

1. Magori K, Legros M, Puente ME, Focks DA, Scott TW, Lloyd AL, et al. Skeeter Buster: a stochastic, spatially explicit modeling tool for studying Aedes aegypti population replacement and population suppression strategies. PLoS Negl Trop Dis. 2009 Sep 1;3(9):e508.

2. Otero M, Solari HG, Schweigmann N. A stochastic population dynamics model for Aedes aegypti: formulation and application to a city with temperate climate. Bull Math Biol. 2006 Nov;68(8):1945–74.

3. Reiner RC, Stoddard ST, Vazquez-Prokopec GM, Astete H, Perkins TA, Sihuincha M, et al. Estimating the impact of city-wide Aedes aegypti population control: An observational study in Iquitos, Peru. PLoS Negl Trop Dis. 2019 May;13(5):e0007255.

4. Focks DA, Haile DG, Daniels E, Mount GA. Dynamic Life Table Model for Aedes aegypti (Diptera: Culicidae): Analysis of the Literature and Model Development. J Med Entomol. 1993 Nov 1;30(6):1003–17.

5. Liebman KA, Stoddard ST, Reiner RC, Perkins TA, Astete H, Sihuincha M, et al. Determinants of Heterogeneous Blood Feeding Patterns by Aedes aegypti in Iquitos, Peru. Barrera R, editor. PLoS Neglected Tropical Diseases. 2014 Feb 13;8(2):e2702.
