# Supplementary material for: Fusing an agent-based model of mosquito population dynamics with a statistical reconstruction of spatio-temporal abundance patterns
Source: PLoS Comput Biol. 2023 Apr 27;19(4):e1010424. doi: 10.1371/journal.pcbi.1010424 (PMC10168549; doi:10.1371/journal.pcbi.1010424)
Supplement: Attachment — Submitted filename: ReviewNotes.pdf [file pcbi.1010424.s018.pdf]

**Fusing an agent-based model of mosquito population dynamics with a statistical reconstruction of spatio-temporal abundance patterns**

As the title suggests, the authors fuse statistical data from a generalized additive model for the spatio-temporal abundance of *Aedes aegypti* mosquitos (derived from real world observations from Iquitos, Peru) with elements of a pre-existing agent-based model for the same species. The work builds on two prior very complex studies, one which developed the generalized additive model (GAM) (reference [19] in the text), and the other which developed an agent-based model (ABM) (reference [26] in the text). The connection between the GAM and the ABM is made via an ordinary differential equation (ODE) model of the life-cycle of the mosquitos, divided into the key developmental life-stages/demographics of eggs, larvae, pupae and adults (I think – as discussed further below, the variables in the fundamentally important system of ODEs are never defined explicitly anywhere in the text, although the parameters in the system are). Within the ODE model there are variable temperature dependent parameters which control the rates at which individuals mature into the next life stage and die. The functions that control the parameter values as a function of environmental temperature/extreme temperature are determined based on prior work in [10]. Key to the study is an additional death rate term, $\mu_c(t)$, applied at the larva and pupa stages, which models death by other mechanisms than those based around temperature. $\mu_c(t)$ is determined via the GAM predications of the number of mosquitoes as a function of space and time in Iquitos and manipulation of the ODE model. Once $\mu_c(t)$ is determined, the ODE model's prediction of the total number of adult mosquitoes mirrors that of the GAM reasonably well. The first three life stages of the mosquitoes and their transitions are also modelled by the first three equations in the system of ODEs within the ABM, and thus $\mu_c(t)$ is an important feature of the ABM that links back to the ODEs and data derived GAM. The ABM constructed also mirrors predictions about the total number of adult mosquitoes from the GAM reasonably well. In a broad sense, $\mu_c(t)$ is a time varying fudge factor that is determined to drive good agreement between the models, but it's use and interpretation here, as a measure of complex mechanisms that are not accounted for elsewhere in the model that may drive mosquito death, seems completely reasonable and valid (provided that $\mu_c(t)$ is greater than or equal to zero). Once the ABM is constructed, some numerical experiments are performed to simulate the effects of two different control strategies on the mosquito population, both of which are applied in the model by modifying/increasing the death rates of mosquitoes over given periods. The model itself seems like a very good tool for investigating the effects of potential control measures in a relatively complex, real world derived scenario, although (as noted by the authors) it can only really be used as a diagnostic rather than prognostic model, as an estimate of $\mu_c(t)$ derived from observational data is required. Given the role that *Ae. aegypti* has in spreading a number of very nasty diseases, such as dengue virus, the model seems like it could be very useful for helping to inform public health initiatives via attempts at controlling the mosquito population. I think the work has a lot of value, but for publication the manuscript needs major revisions, particularly to improve the description of the ODE model, and the vital method for determining $\mu_c(t)$, which I don't think I could understand completely based on the current description in the paper. My detailed comments follow.

**Major Comments**

Deterministic model, pages 11-13. None of the major variables for the system of ODEs, E, L, P and N, are defined explicitly anywhere in the text (including the SI). This needs to be fixed (I think immediately after the system of equations is stated), as it ruins the accessibility of the paper. I interpreted these as the number of eggs (E), larvae (L), pupae (P) and adults, or female adults (N), based on implicit information in later parts of the text; the rest of what I write here is based on this interpretation.

Deterministic model, pages 11-13. Perhaps it would be helpful to tabulate the model parameters in a single table in the main text, with brief notes how each parameter is determined (in concert with the description in the text, and the material provided in the SI).

Deterministic model, starting at the bottom of page 12. I think you could be much more explicit in describing the key process of estimating of $\mu_c(t)$, starting with the first listed step. Perhaps you could introduce some notation for the finite difference approximation to dN/dt based on the GAM derived estimate for N from reference [19] (for example $\Delta N/\Delta t$), and then explicitly write down the equation for $P(\mathbf{x}, t)$ in terms of N and $\Delta N/\Delta t$, derived from equation (4), even though the manipulation is relatively straightforward?

Deterministic model, second step, top of page 13. This step involves numerical solution of equation (1), given the known distribution of $N(\mathbf{x}, t)$ from the GAM in [19] and other specified time/temperature varying parameters using the deSolve package for R. Which numerical integration scheme did you use in deSolve, and why, and what numerical tolerances did you set for the integration? What was the explicit initial condition $E(\mathbf{x}, 0)$ for the numerical integration?

Deterministic model, third and fourth steps, top of page 13. This is where I'm less certain I understand the details of the calculation, and think more detail could be provided. Step 3 involves the reorganisation of equation (3), the equation for dP/dt, to determine L. Given that P was determined at the first step, a numerical estimate for dP/dt could be obtained using finite difference approximations (and if this is what was done, it should be noted explicitly). When L is the subject of equation (3), it depends on the as yet undetermined value of $\mu_c(t)$. Is there an error in equation (3)/should $\mu_c(t)$ appear in that equation? If there is no error, how is L determined when $\mu_c(t)$ is still unknown at this step? (Please provide all the details.) I think there may be an error in step 4 as well, as it's noted that $\mu_c(t)$ is obtained from equation (4), even though $\mu_c(t)$ does not appear in that equation at all. If this is not an error, please provide all the details of the calculation to clarify what other intermediate steps are involved in the calculation.

Deterministic model. Once determined, is $\mu_c(t)$ greater than or equal to zero for all t? If this is so, it might be helpful to include a graph of $\mu_c(t)$, since the parameter itself is vital for the calculations that follow. If not, then interpreting this key parameter as an increased death rate due to complex factors may not be completely correct (because negative values of $\mu_c(t)$ would generate additional population growth).

Agent-based model, page 13. Were only single runs of the ABM performed for each form of calculation (ie. the calibration/validation calculation, numerical simulations of insecticide use, and subsequent investigations on the effects of the order in which spraying was applied across zones)? If so, why (for example, are the calculations time/computationally intensive)? If it is reasonable to perform multiple simulations for each scenario, perhaps it would be

worth doing so? It would then be possible to estimate the mean output from the ABM, along with the variance, and examine related measures, like the probability of eradicating the mosquitos under each of the insecticide regimes (something that cannot be done with the deterministic ODE model), or durations where the mosquito population is below some threshold. Otherwise, it seems like the advantages of the stochastic model are not exploited as much as they could be, especially only with single realisations for each scenario.

Experiments, page 14 and page 18. I think the simulated spraying regimes need to be clarified/explained in greater detail. For example, what constraints did you have in place that led to the duration of the campaigns (27 days in the case of ULV, and 39 days for TIRS). Were the durations informed by real world interventions of this type? I think the ordering in which zones were sprayed should be discussed earlier on in the text as well, since it becomes an element of the discussion later in the text (including on page 19). Perhaps for the base calculations, the numerical zone order for spraying could be listed explicitly.

Results, calibration, page 14. For clarity regarding the calculations for the ODE model, was N (illustrated in Figure 2) determined by integrating the system (1)-(4) after determining $\mu_c(t)$? Why would N determined via the GAM, and N determined by the ODE model after determination of $\mu_c(t)$ differ? This might be addressed through a more detailed explanation of the calculation of $\mu_c(t)$.

Page 19. I think it might be worth investigating what happens on repeat calculations where the order of zones is selected randomly, to see if/show that the hypotheses that the order of zone spraying and persistence of parts of the population are interconnected is well supported.

**Minor Comments**

Author summary, page 7, 5th line of summary. I think the sentence should start "Such models are often categorized as…", rather than "Such models are often categorizes as…".

Author summary, page 8, 1st line. "… when used in concert with an epidemiological model…", rather than "… when used in concert with and epidemiological model…".

Deterministic model, page 12, near the bottom of the page. The sentence starting "This time-varying parameter forms…" needs to be edited/checked.

Agent-based model, page 13, first sentence. The wording of this sentence could be a bit confusing, perhaps because the sentence is too brief. Please make it clear that $\mu_c(t)$ determined using the deterministic model in the current study was then incorporated into an ABM based on that previously used in [26]. I think the current sentence could be misinterpreted as $\mu_c(t)$ having already been used in the previous work described in [26].

Agent-based model, page 13. Are the discrete buildings the only places in space that can be occupied by mosquitoes in the model?

Agent-based model, page 13. The full description of the agent based model in [26] is quite substantial, but to help make the current paper better self-contained, would it be reasonable to include some key details of the ABM in an appendix/SI? A lot of the focus of the paper is on connecting the GAM to the ABM, and then the numerical experiments with the ABM, but there are relatively few details of the ABM itself in the text provided.

Experiments, page 14. The way the first sentence is written could be interpreted as if the spraying strategies were applied in the real world, rather than investigated via simulation. Perhaps you could re-word the first sentence in this section along the lines “We increased death rates over given periods to simulate two insecticide based control strategies…”?

Experiments, page 14. Were only adults subject to increased death rates, or all life stages of the population?

Figure 2 caption, page 15. I think you should include written descriptions of the line colours for the ODE and ABM results, in addition to the description for the GAM results already provided.

Page 15, final paragraph. In the description of Fig. 4, there is reference to the normalisation that was applied (“… normalized by the total abundance that day…”). To clarify in the main text (as this is addressed in the caption for Fig. 4), I think it would be clearer to state that the normalising factor for each day was the total number of adult females across all zones on that day.

Page 16, Fig. 3. Results in subsequent figures, starting with Fig. 4, reference the zones in Fig. 3 via numerical labels. Is it possible to add the zone numbering to Fig. 3, to allow explicit cross referencing with later results, and the discussion around these results?

Page 17, Fig. 4 (and later, similar figures). Would it be informative to include non-normalized analogues of these graphs that just showed the total numbers of females in each zone in the supplementary material?

Page 18, Spatio-temporal effects of spraying. Could these effects also be examined via the ODE model? I think it would be interesting from a modelling point of view to see how much difference the stochastic movement of mosquitos between the buildings/habitats could make compared to the no movement case of the ODE model.

Page 18, Fig. 5. The label on the vertical axis is overwritten on the vertical scale labels.

Page 21, Fig. 7. Would it be better to use the same colour scale as figure 4 here, to make the visual comparison easier? (Perhaps the relatively huge proportion in zone 2 could be represented with some other symbol after the start of spraying?)

Page 26, reference to density dependent population changes for larvae. Perhaps it would be worthwhile referring to the $L^2$ term in equation (2) explicitly here, rather than just the “higher exponent in the density-dependent term…”?

Page 26. I think you should clarify if the “return to baseline observed by Gunning et al. …” was a real-world observational study, or a simulation study.

Page 27. With reference to the statement “as well as produce a realistic response to insecticide applications…”, are there any references that can be added at this point in the text that demonstrate where insecticide use has led to similar dynamics?

Supplementary text (page 33 onward). Should the tables and figures be labelled using the convention SN Table/SN Fig., or Table SN/Fig. SN?

Page 33, S1 Table. I think this table needs to be reformatted a little, so that the line spacing within a variable definition is smaller, and the line spacing between different variables is larger.

Page 36. S2 Fig. The colour scale/colour bar is missing from this figure. In addition, I think the distinction between locations (which I think are specific buildings) and the zones (which contain multiple buildings, I think) might need to be discussed briefly in the caption to explain/clarify why the zones do not all have equal proportions of mosquitos at time zero.
