# Supplementary material for: Fusing an agent-based model of mosquito population dynamics with a statistical reconstruction of spatio-temporal abundance patterns
Source: PLoS Comput Biol. 2023 Apr 27;19(4):e1010424. doi: 10.1371/journal.pcbi.1010424 (PMC10168549; doi:10.1371/journal.pcbi.1010424)
Supplement: Attachment — Submitted filename: response_to_reviewers_20230128.pdf [file pcbi.1010424.s019.pdf]

Response to reviewers

In the below, ordinary text denotes reviewer comments, **bold text our responses**, and red text passages added to the main document**.** Line numbers refer to the corrected version, not the tracked changes version.

Reviewer #1: This manuscript by Cavany et al. reports a new model of Aedes aegypti mosquito population dynamics and its application to vector control. The novelty of the authors' approach is that they use statistically derived estimates of mosquito abundance from survey data and use these estimates to drive the baseline dynamics of a stochastic agent-based model. This latter model, in turn, can be applied to simulate the effects of perturbations to the population – in this case, comparing the effects of two different insecticide spraying regimens across space and time. Overall, this is a strong manuscript that presents a significant advance in vector modeling research, namely, a model that retains faithfulness to empirical data via statistical estimates, while providing predictive power to simulate the effects of interventions or environmental perturbations. My sole criticism is for the authors' to perform more exploration of the parameter space on the effects of ULV and TIRS, to determine how robust their results are across a range of potential parameter values:

We thank the reviewer for their kind words and helpful recommendation. Please see below for a response to your specific comment.

pg. 14: The authors describe how the parameters governing the effects of ULV or TIRS were calibrated to existing data, however it is helpful to demonstrate how robust the results are to variations in these parameter values that may be seen in real-life conditions that do not conform exactly to the cited studies. I recommend the authors perform an exploration of the parameter space by (1) varying the adult mortality rate from ULV and (2) the adult mortality rate and duration of effect from TIRS within a realistic range of values and report how these parameter variations affect mosquito abundance both overall as well as spatially by MOH zone.

Thank you for this suggestion. We added a supplementary plot which shows the distribution by zone following (i) an insecticide treatment with high efficacy (equal to the efficacy of TIRS) but low residuality (equal to that of ULV) and (ii) an insecticide treatment with low efficacy (equal to the efficacy of ULV) but high residuality (equal to that of TIRS). We also added some text describing this new figure (L307-313):

"We also explored the effect of spraying with hypothetical insecticides that had (i) a small effect on mortality (i.e., equal to ULV) but a long residual effect, and (ii) a large effect on mortality (i.e., equal to TIRS) but no residuality (S8 Fig). Scenario (i) (low increase in mortality, high residuality) produced a similar pattern of abundance to that of the TIRS campaign (Fig 7) and scenario (ii) (high increase in mortality, low residuality) produced a pattern similar to the ULV campaign. This suggests that the residual effect of TIRS is more important to its improved overall impact compared to ULV than its larger baseline effect on mortality."

Additional strengths of the manuscript include the clarity of the writing and data presentation.

Thank you.

Reviewer #2:

Fusing an agent-based model of mosquito population dynamics with a statistical reconstruction of spatio-temporal abundance patterns

As the title suggests, the authors fuse statistical data from a generalized additive model for the spatio-temporal abundance of Aedes aegypti mosquitos (derived from real world observations from Iquitos, Peru) with elements of a pre-existing agent-based model for the same species. The work builds on two prior very complex studies, one which developed the generalized additive model (GAM) (reference [19] in the text), and the other which developed an agent-based model (ABM) (reference [26] in the text). The connection between the GAM and the ABM is made via an ordinary differential equation (ODE) model of the life-cycle of the mosquitos, divided into the key developmental life-stages/demographics of eggs, larvae, pupae and adults (I think – as discussed further below, the variables in the fundamentally important system of ODEs are never defined explicitly anywhere in the text, although the parameters in the system are). Within the ODE model there are variable temperature dependent parameters which control the rates at which individuals mature into the next life stage and die. The functions that control the parameter values as a function of environmental temperature/extreme temperature are determined based on prior work in [10]. Key to the study is an additional death rate term, μc(t), applied at the larva and pupa stages, which models death by other mechanisms than those based around temperature. μc(t) is determined via the GAM predications of the number of mosquitoes as a function of space and time in Iquitos and manipulation of the ODE model. Once μc(t) is determined, the ODE model's prediction of the total number of adult mosquitoes mirrors that of the GAM reasonably well. The first three life stages of the mosquitoes and their transitions are also modelled by the first three equations in the system of ODEs within the ABM, and thus μc(t) is an important feature of the ABM that links back to the ODEs and data derived GAM. The ABM constructed also mirrors predictions about the total number of adult mosquitoes from the GAM reasonably well. In a broad sense, μc(t) is a time varying fudge factor that is determined to drive good agreement between the models, but it's use and interpretation here, as a measure of complex mechanisms that are not accounted for elsewhere in the model that may drive mosquito death, seems completely reasonable and valid (provided that μc(t) is greater than or equal to zero). Once the ABM is constructed, some numerical experiments are performed to simulate the effects of two different control strategies on the mosquito population, both of which are applied in the model by modifying/increasing the death rates of mosquitoes over given periods. The model itself seems like a very good tool for investigating the effects of potential control measures in a relatively complex, real world derived scenario, although (as noted by the authors) it can only really be used as a diagnostic rather than prognostic model, as an estimate of μc(t) derived from observational data is required. Given the role that Ae. aegypti has in spreading a number of very nasty diseases, such as dengue virus, the model seems like it could be very useful for helping to inform public health initiatives via attempts at controlling the mosquito population. I think the work has a lot of value, but for publication the manuscript needs major revisions, particularly to improve the description of the ODE model, and the vital method for determining μc(t), which I don't think I could understand completely based on the current description in the paper. My detailed comments follow.

We thank the reviewer for their kind words and helpful recommendations. Please see below for our responses to your specific comments.

Major Comments

Deterministic model, pages 11-13. None of the major variables for the system of ODEs, E, L, P and N, are defined explicitly anywhere in the text (including the SI). This needs to be fixed (I think immediately after the system of equations is stated), as it ruins the accessibility of the paper. I interpreted these as the number of eggs (E), larvae (L), pupae (P) and adults, or female adults (N), based on implicit information in later parts of the text; the rest of what I write here is based on this interpretation.

Thank you for pointing out this mistake, we agree that our oversight severely affects interpretation and have added a description of each of these state parameters (L148):

"where *E* is the number of eggs, *L* is the number of larvae, *P* is the number of pupae, and *N* is the number of female adults."

Deterministic model, pages 11-13. Perhaps it would be helpful to tabulate the model parameters in a single table in the main text, with brief notes how each parameter is determined (in concert with the description in the text, and the material provided in the SI).

We have added this table to the main text (L150):

Table. *Parameter names, definitions, and sources*

**Symbol**	**Definition**	**Source**
n_E	Number of eggs laid per gonotropic cycle	Otero et al. [23]
a	Gonotrophic-cycle rate	Magori et al. [10]
d_E	Development rate of eggs	Magori et al. [10]
d_L	Development rate of larvae	Magori et al. [10]
d_P	Development rate of pupae	Magori et al. [10]
μ_E	Mortality rate of eggs	Magori et al. [10]
μ_L	Mortality rate of larvae	Magori et al. [10]
μ_P	Mortality rate of pupae	Magori et al. [10]
μ_N	Mortality rate of female adults	Magori et al. [10]
μ_C	Other sources of larval and pupal mortality rate	Estimated by fitting deterministic model to estimated larval abundance in Iquitos. Used to fuse the deterministic and statistical models.
κ	Carrying capacity of larvae	Estimated so that density-dependence contributes 90% of deaths on average.

Deterministic model, starting at the bottom of page 12. I think you could be much more explicit in describing the key process of estimating of μc(t), starting with the first listed step. Perhaps you could

introduce some notation for the finite difference approximation to dN/dt based on the GAM derived estimate for N from reference [19] (for example ΔN/Δt), and then explicitly write down the equation for P(x, t) in terms of N and ΔN/Δt, derived from equation (4), even though the manipulation is relatively straightforward?

We have added text to explicitly state how we calculated the finite differences, and for the rearranged equations used to estimate the time series (L178):

- "First, we used equation 4 alongside estimates of $N(t)$ from the statistical model, to obtain estimates of $P(t)$. In this and future steps we found the derivative of a time series (e.g., dN/dt) by taking centered differences (i.e., $\frac{dX}{dt} \simeq \frac{X(t+1) - X(t-1)}{2}$, where X is either E, L, P, or N, and t is the day of the time series) at all time points except the first and last, at which we took forward (i.e., $\frac{dX}{dt} \simeq \frac{X(t+1) - X(t-1)}{2}$) and backward (i.e., $\frac{dX}{dt} \simeq \frac{X(1) - X(0)}{1}$, where n is the number of days in the time series) differences respectively. We could then estimate $P(t)$ from
$$P(x,t) = \frac{\left(\frac{dN}{dt}(x,t) + \mu_N(T_{max}(t))N(x,t)\right)}{d_P(T_W(t))}.$$
- Second, we obtained $E(t)$ by integrating equation 1 using the deSolve package in R."

Deterministic model, second step, top of page 13. This step involves numerical solution of equation (1), given the known distribution of N(x, t) from the GAM in [19] and other specified time/temperature varying parameters using the deSolve package for R. Which numerical integration scheme did you use in deSolve, and why, and what numerical tolerances did you set for the integration? What was the explicit initial condition E(x, 0) for the numerical integration?

We used 12 initial eggs, although the estimate of this time series was insensitive to this initial number of eggs. We obtained this number by integrating the equations for a range of starting conditions and taking the average number of eggs. We used the radau method to solve the integration, and left the numerical tolerances at their default values of $1x10^{-6}$. We have added text to Lxxx in the manuscript to explain this (L187):

"For this and all other integrations we used the radau method, with tolerances kept at their default values of $1x10^{-6}$."

Deterministic model, third and fourth steps, top of page 13. This is where I'm less certain I understand the details of the calculation, and think more detail could be provided. Step 3 involves the reorganisation of equation (3), the equation for dP/dt, to determine L. Given that P was determined at the first step, a numerical estimate for dP/dt could be obtained using finite difference approximations (and if this is what was done, it should be noted explicitly). When L is the subject of equation (3), it depends on the as yet undetermined value of μc(t). Is there an error in equation (3)/should μc(t) appear in that equation? If there is no error, how is L determined when μc(t) is still unknown at this step? (Please provide all the details.) I think there may be an error in step 4 as well, as it's noted that μc(t) is obtained from equation (4),

even though μc(t) does not appear in that equation at all. If this is not an error, please provide all the details of the calculation to clarify what other intermediate steps are involved in the calculation.

You are correct, and there were several errors in our original description of this process. Apologies for this oversight. We have now rewritten steps three and four to clarify these errors. Briefly, step 3 should have explained that equation 2 was rearranged to make μ_c the subject, then this was inserted into equation 3, yielding a first-order differential equation in L with known coefficients. We then solve this equation numerically to yield L. We can then obtain μ_c from equation 2 (or 3) (L189):

"Third, we obtained $L(t)$ by combining equations 2 and 3 to remove μ_c from them, yielding a first-order differential equation in L, which we solve numerically using deSolve:

$$\frac{dX}{dt} \simeq \frac{X(n) - X(n-1)}{1}.$$

Finally, we obtained $P(x,t) = \frac{\left(\frac{dN}{dt}(x,t) + \mu_N(T_{max}(t))N(x,t)\right)}{d_P(T_W(t))}$ by rearranging equation 2 to obtain

$$L(x,t) = d_E E + \left(\frac{1}{P}\frac{dP}{dt} + d_P + \mu_P - d_L - \mu_L\right)L - \frac{d_L L^2}{P} - \frac{L^{\alpha+1}}{\kappa}."$$

Deterministic model. Once determined, is μc(t) greater than or equal to zero for all t? If this is so, it might be helpful to include a graph of μc(t), since the parameter itself is vital for the calculations that follow. If not, then interpreting this key parameter as an increased death rate due to complex factors may not be completely correct (because negative values of μc(t) would generate additional population growth).

We have now included the time series of this parameter as a supplementary figure (S1 Fig), and can confirm that it is never negative.

Agent-based model, page 13. Were only single runs of the ABM performed for each form of calculation (ie. the calibration/validation calculation, numerical simulations of insecticide use, and subsequent investigations on the effects of the order in which spraying was applied across zones)? If so, why (for example, are the calculations time/computationally intensive)? If it is reasonable to perform multiple simulations for each scenario, perhaps it would be worth doing so? It would then be possible to estimate the mean output from the ABM, along with the variance, and examine related measures, like the probability of eradicating the mosquitos under each of the insecticide regimes (something that cannot be done with the deterministic ODE model), or durations where the mosquito population is below some threshold. Otherwise, it seems like the advantages of the stochastic model are not exploited as much as they could be, especially only with single realisations for each scenario.

We ran multiple runs of the ABM, but to simplify the exposition only showed single runs. In this model, the mosquito component is largely deterministic in its behavior, and so running more simulations adds little. This is likely because of the large population size (~10^5), the deterministic treatment of the immature stages, and the fact that the time series is strongly forced by μ_c. We added some text to describe this, and added a supplementary figure to show the lack of variability in the mosquito population time series (L215).

"Each of the main text plots showing the mosquito time series output from the ABM show a single simulation. In this model, the mosquito population dynamics at the city-level do not appear very stochastic. This is likely because of the large population size (~10^5), the deterministic treatment of the immature stages, and the fact that the time series is strongly forced by μ_c. We show the lack of variability across 400 simulations of the ABM in S2 Fig."

Experiments, page 14 and page 18. I think the simulated spraying regimes need to be clarified/explained in greater detail. For example, what constraints did you have in place that led to the duration of the campaigns (27 days in the case of ULV, and 39 days for TIRS). Were the durations informed by real world interventions of this type? I think the ordering in which zones were sprayed should be discussed earlier on in the text as well, since it becomes an element of the discussion later in the text (including on page 19). Perhaps for the base calculations, the numerical zone order for spraying could be listed explicitly.

Yes, the spraying protocols were based in part on previous spraying campaigns in Iquitos. We added text on LXXX in the methods to describe this in more detail (L228 & L233):

"The city-wide ULV campaign typically took around 27 days to complete, and consisted of three rounds during which each was sprayed once with probability 0.7. The length of the campaign and the probability that a house was sprayed in a given round were chosen to reflect past ULV spraying campaigns in Iquitos, Peru, in which an average of 11,000 houses are sprayed per day."

"City-wide TIRS campaigns took around 39 days to complete, and consisted of just one round, with the same probability of 0.7 that an individual house is sprayed. This length was based on the observation that TIRS takes ~5 times as long to apply as ULV [29], and amounted to 2,000 houses being sprayed per day."

We also added numbering to figure 3 to show the spraying order of zones, and some text to point to this figure (L238):

"Zones were sprayed in ascending order according to the numbers displayed in Fig 3.**"**

Results, calibration, page 14. For clarity regarding the calculations for the ODE model, was N (illustrated in Figure 2) determined by integrating the system (1)-(4) after determining μc(t)?

Yes, we have now clarified this in the text (L243):

"Here the ODE model results were obtained by integrating equations 1-4 using the derived $\mu_c(t)$."

Why would N determined via the GAM, and N determined by the ODE model after determination of μc(t) differ? This might be addressed through a more detailed explanation of the calculation of μc(t).

This is due to the fact that all parameters must be non-negative and the discretization of the ODE system. We added text to explain this (L246).

"The ODE model does not match the GAM perfectly due to the fact that all parameters were forced to be non-negative and the discretization of the ODE system, both of which cause small discrepancies to be introduced."

Page 19. I think it might be worth investigating what happens on repeat calculations where the order of zones is selected randomly, to see if/show that the hypotheses that the order of zone spraying and persistence of parts of the population are interconnected is well supported.

As the purpose of this paper was largely to develop the methodological approach, we felt that more simulations may have been too far removed from this focus. However, as the hypothesis you mention did not have sufficient support, we have removed it from the text.

Minor Comments

Author summary, page 7, 5th line of summary. I think the sentence should start "Such models are often categorized as...", rather than "Such models are often categorizes as...".

We have made this change.

Author summary, page 8, 1st line. "... when used in concert with an epidemiological model...", rather than "... when used in concert with and epidemiological model...".

We have made this change.

Deterministic model, page 12, near the bottom of the page. The sentence starting "This time-varying parameter forms..." needs to be edited/checked.

We have updated this sentence to the following (L173):

"Calculation of this time-varying parameter is the key step in our approach, because it is calibrated to spatiotemporal estimates of mosquito abundance in Iquitos during 2000-10 by Reiner et al. [19], and it enabled us to account for differences between those estimates and the ODE."

Agent-based model, page 13, first sentence. The wording of this sentence could be a bit confusing, perhaps because the sentence is too brief. Please make it clear that μc(t) determined using the deterministic model in the current study was then incorporated into an ABM based on that previously used in [26]. I think the current sentence could be misinterpreted as μc(t) having already been used in the previous work described in [26].

We have updated this sentence to the following (L199):

"We incorporated the $\mu_c(t)$ time series obtained from manipulation of the ODE model into an ABM of DENV transmission based on the one previously used in Perkins et al [26]."

Agent-based model, page 13. Are the discrete buildings the only places in space that can be occupied by mosquitoes in the model?

Yes, mosquitoes only exist in buildings. When they decide to move, they essentially move instantaneously to a nearby building. We have added text to describe this (L212):

"Mosquitoes of all stages only exist in the model within buildings."

Agent-based model, page 13. The full description of the agent based model in [26] is quite substantial, but to help make the current paper better self-contained, would it be reasonable to include some key details of the ABM in an appendix/SI? A lot of the focus of the paper is on connecting the GAM to the ABM, and then the numerical experiments with the ABM, but there are relatively few details of the ABM itself in the text provided.

We have added some supplementary text (S2 Text) to describe the ABM in more detail, and made reference to this at the end of the Agent-based model section in the main text.

Experiments, page 14. The way the first sentence is written could be interpreted as if the spraying strategies were applied in the real world, rather than investigated via simulation. Perhaps you could re-word the first sentence in this section along the lines "We increased death rates over given periods to simulate two insecticide based control strategies..."?

We changed this sentence to be the following (L221):

"We examined the effect of spraying with insecticide via numerical simulation, with either an instantaneous effect (ultra-low volume spraying; ULV) or a residual effect (targeted insecticide residual spraying; TIRS)."

Experiments, page 14. Were only adults subject to increased death rates, or all life stages of the population?

Only adults were subject to the increased mortality rates, we have added text to described this in more detail (L225 & L232):

"There was no effect on the mortality of the immature stages."

"As for ULV, there was no effect on the mortality of the immature stages."

Figure 2 caption, page 15. I think you should include written descriptions of the line colours for the ODE and ABM results, in addition to the description for the GAM results already provided.

We have made this change (L258):

"… , the purple line shows those predicted by the ODE model, and the pink line those predicted by the ABM."

Page 15, final paragraph. In the description of Fig. 4, there is reference to the normalisation that was applied ("... normalized by the total abundance that day..."). To clarify in the main text (as this is addressed in the caption for Fig. 4), I think it would be clearer to state that the normalising factor for each day was the total number of adult females across all zones on that day.

We have changed this description to the following (L264):

"Fig 4 shows the normalized abundance in each of 35 Ministry of Health (MoH) zones every hundred days, where the normalizing factor for each day was the total number of adult females across all zones on that day."

Page 16, Fig. 3. Results in subsequent figures, starting with Fig. 4, reference the zones in Fig. 3 via numerical labels. Is it possible to add the zone numbering to Fig. 3, to allow explicit cross referencing with later results, and the discussion around these results?

We have made this change.

Page 17, Fig. 4 (and later, similar figures). Would it be informative to include non-normalized analogues of these graphs that just showed the total numbers of females in each zone in the supplementary material?

We have now included these figures in the supplementary material. These are figures S3 Fig (no spraying), S4 Fig (ULV spraying), and S5 Fig (TIRS spraying).

Page 18, Spatio-temporal effects of spraying. Could these effects also be examined via the ODE model? I think it would be interesting from a modelling point of view to see how much difference the stochastic movement of mosquitos between the buildings/habitats could make compared to the no movement case of the ODE model.

This is an interesting question. However, we refrained from doing this in the study, as the purpose here was not to develop the ODE model as a stand-alone model, but rather to use it as a means to calibrate the agent-based model to the statistical model.

Page 18, Fig. 5. The label on the vertical axis is overwritten on the vertical scale labels.

We have amended this.

Page 21, Fig. 7. Would it be better to use the same colour scale as figure 4 here, to make the visual comparison easier? (Perhaps the relatively huge proportion in zone 2 could be represented with some other symbol after the start of spraying?)

This is a good idea, but we felt that the version with the wider color scale was also informative of the scale of the spatial heterogeneity introduced following TIRS, so we instead made Fig 7 as a

two panel plot – one panel with the wider color scale, and one with the same color scale used in Fig 4.

Page 26, reference to density dependent population changes for larvae. Perhaps it would be worthwhile referring to the L2 term in equation (2) explicitly here, rather than just the “higher exponent in the density-dependent term...”?

We have made this change.

Page 26. I think you should clarify if the “return to baseline observed by Gunning et al. ...” was a real-world observational study, or a simulation study.

We have clarified this.

Page 27. With reference to the statement “as well as produce a realistic response to insecticide applications...”, are there any references that can be added at this point in the text that demonstrate where insecticide use has led to similar dynamics?

We added the following four references to this statement:

27. Gunning CE, Okamoto KW, Astete H, Vasquez GM, Erhardt E, Del Aguila C, et al. Efficacy of Aedes aegypti control by indoor Ultra Low Volume (ULV) insecticide spraying in Iquitos, Peru. Barrera R, editor. PLOS Neglected Tropical Diseases. 2018;12: e0006378. doi:10.1371/journal.pntd.0006378

30. Perich MJ, Rocha N O, Castro A L, Alfaro A W, Platt KB, Solano T, et al. Evaluation of the efficacy of lambda-cyhalothrin applied by three spray application methods for emergency control of Aedes aegypti in Costa Rica. J Am Mosq Control Assoc. 2003;19: 58–62.

31. Perich MJ, Sherman C, Burge R, Gill E, Quintana M, Wirtz RA. Evaluation of the efficacy of lambda-cyhalothrin applied as ultra-low volume and thermal fog for emergency control of Aedes aegypti in Honduras. J Am Mosq Control Assoc. 2001;17: 221–224.

32. Horstick O, Runge-Ranzinger S, Nathan MB, Kroeger A. Dengue vector-control services: how do they work? A systematic literature review and country case studies. Trans R Soc Trop Med Hyg. 2010;104: 379–386. doi:10.1016/j.trstmh.2009.07.027

Supplementary text (page 33 onward). Should the tables and figures be labelled using the convention SN Table/SN Fig., or Table SN/Fig. SN?

PLOS journals require the former, so we have used that for this submission.

Page 33, S1 Table. I think this table needs to be reformatted a little, so that the line spacing within a variable definition is smaller, and the line spacing between different variables is larger.

We have made these changes.

Page 36. S2 Fig. The colour scale/colour bar is missing from this figure. In addition, I think the distinction between locations (which I think are specific buildings) and the zones (which contain multiple buildings, I think) might need to be discussed briefly in the caption to explain/clarify why the zones do not all have equal proportions of mosquitos at time zero.

We have added a color bar and clarified this.

Reviewer #3: In “Fusing an agent-based model of mosquito population dynamics with a statistical reconstruction of spatio-temporal abundance patterns”, Cavany et al present a novel method for parameterizing an agent based model of mosquito dynamics based on household survey data from Iquitos, Peru. While their method requires a number of intermediate steps that are not always trivial, evident by their first step having been published on its own, it is likely still preferable to current methods of parameterizing ABMs, e.g. approximate-bayesian computation. Overall, the paper is well written and easy to follow. However, there are some minor comments that I believe should be addressed before publication.

We thank the reviewer for their kind words and helpful recommendations. Please see below for our responses to your specific comments.

• In the methods for the deterministic model, I am confused by the third and final steps. µc(t) is not in equation 4 and why would L(t) not be obtained from equation 2 (d(L(t))/dt)? I am not sure if this is simply a numbering issue or if I am missing something, but it needs clarification.

You are correct, and there were several errors in our original description of this process. Apologies for this oversight. We have now rewritten steps three and four to clarify these errors. Briefly, step 3 should have explained that equation 2 was rearranged to make μ_c the subject, then this was inserted into equation 3, yielding a first-order differential equation in L with known coefficients. We then solve this equation numerically to yield L. We can then obtain μ_c from equation 2 (or 3). This is explained in L189 onwards:

“Third, we obtained *L*(*t*) by combining equations 2 and 3 to remove μ_c from them, yielding a first-order differential equation in L, which we solve numerically using deSolve:

$$\mu_c(t).$$

Finally, we obtained $\mu_c(t)$ by rearranging equation 2 to obtain

$$\mu_c(t)=\frac{\left(d_E(T_W(t))E(x,t)-\frac{dL}{dt}(x,t)+\left(d_L(T_W(t))+\mu_L(T_{W,max}(t))+\frac{L^2(x,t)}{\kappa(x)}\right)L(x,t)\right)}{L(x,t)}.”$$

• In the methods for the experiments, I am not sure what to make of the increased mortality due to ULV and residual spraying. Typically, mortality rates have a /day unit. Does this mean that there are 1.5 (or 9) additional deaths per day regardless of population size? If so, what is done to keep population sizes above 0? Or is this perhaps the number of additional deaths assuming the mean or equilibrium population size?

As this describes a mortality rate rather than a risk, it is possible for the mortality rate to be above 1 without implying that the population size must go below 0. In the model, we convert the daily rate, after adjusting for any insecticide application, to a daily probability of death according to probability = 1 – exp(-rate). We have expanded on this in the text (L226):

"In the agent-based model, the daily mortality rate is converted into a daily probability of death according to $\mu_c(t)$."

• What is the justification for applying the residual spray to every household in the city? This does not seem like a realistic choice. At the bare minimum, the authors should discuss the number of households that could be reasonably expected to be treated in a given period.

We actually assumed that houses are sprayed with probability 0.7, and have now added some text to clarify this. We also explain the justification for the time taken for a TIRS campaign, which amounts to 2,000 houses being sprayed per day (L225 onwards). Both of these are derived from past spraying campaigns and published data.

"There was no effect on the mortality of the immature stages. In the agent-based model, the daily mortality rate is converted into a daily probability of death according to $prob = 1 - \exp(-rate)$. The city-wide ULV campaign, typically took around 27 days to complete, and consisted of three rounds during which each was sprayed once with probability 0.7. The length of the campaign and the probability that a house was sprayed in a given round were chosen to reflect past ULV spraying campaigns in Iquitos, Peru, in which an average of 11,000 houses are sprayed per day. TIRS increased the adult mortality rate by 9 deaths/day and lasted for 90 days, after which the effect decayed exponentially [28]. As for ULV, there was no effect on the mortality of the immature stages. City-wide TIRS campaigns took around 39 days to complete, and consisted of just one round, with the same probability of 0.7 that an individual house is sprayed. This length was based on the observation that TIRS takes ~5 times as long to apply as ULV [29], and amounted to 2,000 houses being sprayed per day."

• When comparing the ABM to the ODE and GAM, the authors should mention how many runs of the ABM are being averaged over. Related to this, is the trajectory given for the ABM in figure 2 a mean trajectory or from an individual run? It would also be helpful to include some mention of how much variation is seen between model runs.

We ran multiple runs of the ABM, but to simplify the exposition only showed single runs. In this model, the mosquito component is largely deterministic in its behavior, and so running more simulations adds little. This is likely because of the large population size (~10^5), the deterministic treatment of the immature stages, and the fact that the time series is strongly forced by μ_c. We added some text to describe this, and added a supplementary figure to show the lack of variability in the mosquito population time series (L215).

“Each of the main text plots showing the mosquito time series output from the ABM show a single simulation. In this model, the mosquito population dynamics at the city-level do not appear very stochastic. This is likely because of the large population size (~10^5), the deterministic treatment of the immature stages, and the fact that the time series is strongly forced by μ_c. We show the lack of variability across 400 simulations of the ABM in S2 Fig.”

• If the trajectory in Figure 2 is from an individual run, it seems that the ABM, as well as the ODE, end up smoothing out much of the variation that is apparent in the GAM. This would be fine if that variation is not biologically relevant or representative of actual changes in the population, e.g. measurement error, but I do not expect this is the case. I think this is something the authors should discuss. If the trajectory shown is in fact a mean trajectory, it would be beneficial to know how a single run compares to the GAM.

The smoothing out of this day-to-day variation is likely a consequence of such large fluctuations being incommensurate with the slower population dynamics described by the mechanistic models (at least without having unrealistic or negative parameter values). We added some text to discuss this observation (L378):

“The second exception is that both the ODE model and the ABM smooth out some of the day-to-day variability in mosquito abundance predicted by the GAM. This is likely a consequence of the sometimes large day-to-day fluctuations in abundance predicted by the GAM being incommensurate with the slower population dynamics described by the mechanistic model. The GAM can accommodate these larger fluctuations as the included environmental predictor variables vary substantially from day-to-day. It is also worth noting that the fact that our mechanistic model cannot recreate the full extent of the GAM’s variability may indicate that some of these larger day-to-day fluctations may not be actually physically possible.”

• In general, the manuscript would benefit from expanded figure captions that include the take-away message. For some of the figures (i.e. Figure 6), I am not sure what I am supposed to see.

We have added a take-home message in bold to most figures. For example, for Fig 7 (formerly Fig 6), we included the following (L324):

“TIRS has a substantial effect on the spatial distribution of abundance for most of the year following spraying, as some zones are reduced to zero abundance.”

• In the results on the mosquito age distribution following spraying. I am confused about the last sentence of this paragraph, “Occasionally, such as near the start of 2004, a cohort of adult mosquitoes survived longer and the age distribution became less skewed and sometimes bimodal”. What is happening here? Is this just a result of the stochasticity of the model at low population sizes? Does it show up across multiple simulations or is this a result that is only seen in a single or a few simulations.

Again, it would be good to know if these results are from several simulation runs or only a single simulation.

This is likely a consequence of the large drop in abundance around that time. We have attempted to describe this in greater detail (L335):

"This is likely a consequence of the precipitous drop in abundance around this time necessitating a large value of $\mu_c(t)$. This in turn leads to a temporarily large drop in the total population of larvae and pupae and hence a 'missing' cohort of adult mosquitoes and a bimodal age distribution."

• The authors should include a justification for why only Tmax is used for estimating mortality rates. How would using another measure affect the results?

The functions we used to describe mortality rates were functions of both minimum and maximum temperatures, but in practice the daily minimum temperatures were never low enough to affect mortality so we simply wrote them as a function of maximum temperature. The functions we used for all parameters had threshold effects for both minimum (and maximum) temperature, above (or below) which the minimum (or maximum) temperature did not affect mortality. We explain this in more detail in the text (L156):

"Although the expressions describing these parameters are functions of both daily minimum and maximum temperatures (S1 Text), in the period 2000-2010 the daily minimum temperature in Iquitos never went below the threshold at which minimum temperature affected mortality, so we write these parameters as functions of T_{max} only."

• Since the thermal response curves used are justified based on the fact "the temperature never gets cold enough to cause mortality from cold temperatures", the authors should include at least summary information on the temperatures observed in Iquitos. The authors should also justify the use of Magori et al and Otero et al over more recent publications, e.g. Mordecai et al. 2017, which estimated thermal response curves for many of these parameters based on experimental data. Especially when the thermal responses presented in Mordecai et al would not necessitate the temperature not falling below the optimal temperature for mosquito mortality, which I doubt.

We have included a figure showing the temperature time series in Iquitos (S10 Fig) and some text pointing to this (L491).

"See S11 Fig for the temperature time series for air and water in Iquitos in the period 2000-2010."

As our model was based in part on Magori et al. and Otero et al., we wanted to use parameters from those papers to retain comparability with them. Moreover, Mordecai et al does not contain all of the necessary parameters needed for our model (in particular, development and mortality rates for each immature stage). We added some text to justify this choice (L468):

“We parameterize the transitions in the early life-stages in a way analagous to that of Magori et al. Although there are more recent estimates of the thermal responses of *Aedes aegypti* life traits, such as Mordecai et al. [34], we wished to retain comparability with prior agent-based models of *Ae. aegypti* population dynamics and so used the same parameterization as Magori et al.”

• Figure S1, what is the unit on change in mortality rate? Is this an absolute change (/day) or a relative change?

We have added the units to this graph.

• The authors should discuss how this method compares to other ways of calibrating ABM to this type of data.

We added some discussion of the sequential Monte Carlo approach for fitting stochastic models (L404):

“Another alternative to our approach would have been to directly calibrate an agent-based population dynamics model to the household mosquito survey from Iquitos using a sequential Monte Carlo approach [31] or approximate Bayesian computation [32]. The former approach can be computationally very costly for a complex model such as this, and our approach was comparatively simpler. Moreover, by fusing mechanistic and statistical models, our approach was able to leverage data on environmental covariates that may have been difficult to incorporate in a fully-mechanistic framework given the number of parameters and complex functional relationships that might entail.”

Typos and miscellaneous:
Abstract: yellow virus should be yellow fever virus

We have made this change.

Figure 2: The line is described as blue. It appears to be purple. Is the fact that the trajectory lines extend beyond the x-axis an intentional choice?

We have clarified the colors in the caption, and extended the *x*-axis line to the end of the plot (L256).

“Fig 2: City-wide female adult Ae. aegypti abundance in Iquitos, as predicted by the three models. The periwinkle blue line shows daily values of abundance predicted by the GAM, the purple line shows those predicted by the ODE model, and the pink line those predicted by the ABM. GAM: generalized additive model; ODE: ordinary differential equation model; ABM: agent-based model.”

Figure 5: the y-axis title and label text are overlapping

We have corrected this.
