# Supplementary material for: Fusing an agent-based model of mosquito population dynamics with a statistical reconstruction of spatio-temporal abundance patterns
Source: PLoS Comput Biol. 2023 Apr 27;19(4):e1010424. doi: 10.1371/journal.pcbi.1010424 (PMC10168549; doi:10.1371/journal.pcbi.1010424)
Supplement: Attachment — Submitted filename: ReviewNotes_Revision1.pdf [file pcbi.1010424.s020.pdf]

Fusing an agent-based model of mosquito population dynamics with a statistical reconstruction of spatio-temporal abundance patterns – revision 1

My apologies to the authors and the editors for the delay in returning my comments for this revision, I am very sorry about this. I have a substantive service role as part of my academic duties, and this has restricted my ability to work on my review over a large portion of the last month.

As with my previous review of the work, I think this is a very interesting and worthwhile study. The authors have made a very substantial effort to answer all my questions, and that of the other reviewers, and in the process have made substantial revisions to the paper itself. I very much appreciate the effort that the authors made in revising, correcting and supplementing their description of the process involved for calculating $\mu_c(t)$, it is so much clearer from my perspective, as is the overall model description. I hope the details included help make the article more accessible to a technically engaged readership. Beyond my thoughts about the interest of the paper, and the improvements in presenting the model, I only have a few other minor comments, as follows:

Line 66, Author Summary, I think the portion of text written as "… in concert with and epidemiological model…" should be "… in concert with an epidemiological model…".

Line 162, near the end of the line, the word was is italicised (and I don't think that it should be).

Lines 171, 196, 198, 207 (and maybe others). Some spacing seems to be missing from the text when $\mu_c(t)$ is referenced.

Lines 174 to 176. Rather than referring to "this time-varying parameter" in this sentence, perhaps it would be better refer to μ_c directly, to avoid any ambiguity about the parameter being discussed?

Finite difference approximations (Lines 180, 182). I think this may be a matter of personal preference largely, but would it be better to write the approximations as:

$$\frac{dX}{dt} \simeq \frac{X(t+\Delta t) - X(t-\Delta t)}{2\Delta t}, \quad \frac{dX}{dt} \simeq \frac{X(t+\Delta t) - X(t)}{\Delta t}, \text{ and } \frac{dX}{dt} \simeq \frac{X(t) - X(t-\Delta t)}{\Delta t},$$

where Δt is the duration between consecutive time steps (rather than having the time steps written in units of 1s)? Along with this suggestion, I have a related question – was the discrete time step really 1 unit, and what were the underlying units (seconds, minutes, hours)?

Line 190 (equation). Should the last term on the right hand side of this equation be $-\frac{L^3}{\kappa}$, rather than $-\frac{L^{\alpha+1}}{\kappa}$?

Line 200 to 201. The model description in the S2 text contains quite a lot of details about the human and mosquito agents in the models, however the sentences across these two lines suggest "These features were not used in the present study.". Could you please clarify which features are referred to here, as it seems they are the humans and mosquitos discussed in quite

a lot of detail in S2 text? (Perhaps it doesn’t make sense to detail the human and mosquito agents in S2, if they are not used for the study?)
