# Supplementary material for: Fusing an agent-based model of mosquito population dynamics with a statistical reconstruction of spatio-temporal abundance patterns
Source: PLoS Comput Biol. 2023 Apr 27;19(4):e1010424. doi: 10.1371/journal.pcbi.1010424 (PMC10168549; doi:10.1371/journal.pcbi.1010424)
Supplement: Attachment — Submitted filename: response_to_reviewers_rd2_20230330.pdf [file pcbi.1010424.s021.pdf]

Response to reviewers

In the below, ordinary text denotes reviewer comments, **bold text our responses**, and red text passages added to the main document**.** Line numbers refer to the corrected version, not the tracked changes version.

Reviewer #1: The authors have fully addressed all my critiques.

Thank you for your feedback on the paper

Reviewer #2: My apologies to the authors and the editors for the delay in returning my comments for this revision, I am very sorry about this. I have a substantive service role as part of my academic duties, and this has restricted my ability to work on my review over a large portion of the last month.

As with my previous review of the work, I think this is a very interesting and worthwhile study. The authors have made a very substantial effort to answer all my questions, and that of the other reviewers, and in the process have made substantial revisions to the paper itself. I very much appreciate the effort that the authors made in revising, correcting and supplementing their description of the process involved for calculating $\mu_c(t)$, it is so much clearer from my perspective, as is the overall model description. I hope the details included help make the article more accessible to a technically engaged readership. Beyond my thoughts about the interest of the paper, and the improvements in presenting the model, I only have a few other minor comments, as follows:

Thank you for your kind words and additional feedback. Please see below for our responses to your comments.

Line 66, Author Summary, I think the portion of text written as "… in concert with and epidemiological model…" should be "… in concert with an epidemiological model…".

We have made this change.

Line 162, near the end of the line, the word was is italicised (and I don't think that it should be).

We have un-italicized 'was'

Lines 171, 196, 198, 207 (and maybe others). Some spacing seems to be missing from thetext when $\mu_c(t)$ is mentioned.

We have added spacing to these and other similar typographical errors.

Lines 174 to 176. Rather than referring to "this time-varying parameter" in this sentence, perhaps it would be better to refer to $\mu_c(t)$ directly, to avoid any ambiguity about the parameter being discussed?

We have made this change.

Finite difference approximations (Lines 180, 182). I think this may be a matter of personal preference largely, but would it be better to write the approximations as:

$$\frac{dX}{dt} \simeq \frac{X(t+\Delta t) - X(t-\Delta t)}{2\Delta t}, \quad \frac{dX}{dt} \simeq \frac{X(t+\Delta t) - X(t)}{\Delta t}, \text{ and } \frac{dX}{dt} \simeq \frac{X(t) - X(t-\Delta t)}{\Delta t},$$

where Δt is the duration between consecutive time steps (rather than having the time steps written in units of 1s)? Along with this suggestion, I have a related question – was the discrete time step really 1 unit, and what were the underlying units (seconds, minutes,hours)?

In this case, as we are estimating these time series based on time series of daily temperatures, we are limited in this case to time steps of 1 day (or multiple days). With this in mind, and to avoid introducing additional notation (Δ*t*), we have chosen to keep these expressions as they were. We amended the text slightly to make it clear that *t* is measured in days.

Line 190 (equation). Should the last term on the right hand side of this equation be $-L^3/\kappa$, rather than $-L^{\alpha+1}/\kappa$?

Yes, you are correct, apologies for this mistake. We have corrected this in the text.

Line 200 to 201. The model description in the S2 text contains quite a lot of details about the human and mosquito agents in the models, however the sentences across these two lines suggest "These features were not used in the present study.". Could you please clarify which features are referred to here, as it seems they are the humans and mosquitos discussed in quite a lot of detail in S2 text? (Perhaps it doesn't make sense to detail the human and mosquito agents in S2, if they are not used for the study?)

We amended this sentence to clarify the components which were not used in the study:

"These human and virus transmission components were not used in the present study."

We also removed much of the extraneous detail in S2 Text.

Reviewer #3: The manuscript “Fusing an agent-based model of mosquito population dynamics with a statistical reconstruction of spatiotemporal abundance patterns” has been much improved following revisions and the authors have addressed all of my concerns. The paper is now very clearly written and the methods are easily understood. My opinion is that the methods described in this manuscript will be useful for the development of future models of mosquito control. I have a few very minor comments that I believe further improve the manuscript.

Thank you for your kind words and additional feedback.

• On line 89, the authors describe estimating population abundance as being “typically” done based on MMR. While I agree that it is often done this way, I feel that it more often done based on trap data due to the ease and availability of trap data.

We have amended this text to clarify this:

“…, typically based on trap data or mark-release-recapture experiments.”

• On lines 335-336 the authors state “This is likely a consequence of the precipitous drop in abundance around this time necessitating a large value of μc(t).” Do the authors believe this to be a numeric relic from the fitting process or reflective of something biological? If it is biological, do the authors have any theories as to what caused it and its likely impacts?

It is unclear whether this is an artifact of the statistical model, or due to a real event causing the drop, and both are plausible. There are several plausible events which could cause this drop, such as flooding events or control measures. We now discuss this in the text:

“This is likely a consequence of the precipitous drop in abundance around this time necessitating a large value of $\mu_c(t)$, This abrupt drop in abundance could be due to a physical event, for instance a larval habitat reduction campaign or a flooding event flushing out containers, or it cold be an artifact of the statistical model fit.”

• On lines 383-385 the authors make the comment “It is also worth noting that the fact that our mechanistic model cannot recreate the full extent of the GAM’s variability may indicate that some of these larger day-to-day fluctuations may not be physically possible.” I think this is an extremely good point that the authors could expand further on. Also fluctuations is misspelled in the text.

We have expanded on this slightly in the discussion, in particular making reference to your previous comment:

“In particular, large changes in the environmental variables could lead to large changes in the abundance predicted by the statistical model, but that were not reflected in reality. Alternatively, the fact that the mechanistic model cannot recreate the GAM’s variability could suggest there are additional processes taking places which are not able to captured by $\mu_c(t)$.”
